# Reliability of quartz-hosted melt inclusions and discrete rhyolite reservoirs revealed by H isotopes, trace elements, and volatiles

**DOI:** 10.1126/sciadv.adw1667

**Published:** 2026-03-13

**Authors:** Sarah M. Hickernell, Ayla S. Pamukçu, Brian D. Monteleone

**Affiliations:** ^1^ Earth & Planetary Sciences, Stanford University, Stanford, CA 94305, USA.; ^2^ Woods Hole Oceanographic Institution, Woods Hole, MA 02543, USA.

## Abstract

High-silica magmas (≥68 weight % silicon dioxide) have produced some of the largest and most impactful volcanic eruptions in Earth’s history. Quartz-hosted melt inclusions can provide critical insights into the architecture, evolution, and eruption of silicic magmas; however, the reliability of mineral-hosted melt inclusion compositions has been questioned because of potential postentrapment modification of inclusions via diffusive exchange. We demonstrate that with careful sample selection, both naturally glassy and experimentally homogenized quartz-hosted melt inclusions can retain meaningful preeruptive signatures and provide useful insights into preeruptive magmatic conditions. We also show that hydrogen isotope measurements and detailed descriptions of melt inclusion geometries are particularly critical for identifying postentrapment diffusive exchange in melt inclusions, whether it is due to natural or experimental processes. Last, combining data from measurements of quartz-hosted melt inclusion hydrogen isotopes, trace elements, volatiles, and geometries, we fingerprint multiple discrete rhyolite magma reservoirs in the Highland Range volcanic sequence (NV, USA).

## Teaser

Meaningful natural signatures in quartz-hosted melt inclusions identified via characterization of size, H isotopes, and volatiles.

## INTRODUCTION

Many of the largest, most hazardous, and most explosive eruptions in Earth’s history have involved high-silica magmas [≥68 wt % SiO_2_; e.g., Yellowstone (*1*) and Bishop Tuff (*2*)]. Although eruptions of such magmas have been infrequent in recorded history, the widespread deposits of rhyolites and dacites in the geologic record enable study of these hazardous magmatic systems. Characterizing and understanding the generation, storage, evolution, and eruption of silicic magmas in the past are fundamental to improving future monitoring and hazard mitigation efforts at active and high-threat silicic volcanoes.

Magmatic volatile species (H_2_O, CO_2_, Cl, F, and S) play a major role in many aspects of magma evolution and eruption. They can control the explosivity of eruptions (*3*), which is particularly important for high-viscosity silicic magmas. Magmatic volatiles are also important contributors to the volatile budget of the atmospheres of Earth and other planets (*4*). The initial dissolved volatile contents of magmas and their isotopic signatures are dictated by their source (*5*, *6*), and magmatic volatiles evolve with and drive magma cooling, crystallization, and differentiation (*7*, *8*). Volatile solubilities in magmas are also largely a function of pressure (*9*), and geobarometers based on magmatic H_2_O and CO_2_ are a widely used tool for exploring the depths and distributions of magma reservoirs (*9*–*11*). These examples underscore the importance of effectively characterizing the preeruptive volatile contents of magmas for our understanding of the architecture, physical and compositional evolution, and eruption of magmatic systems.

Glassy melt inclusions—parcels of crystal-free melt entrapped during crystal growth and quenched to glass upon eruption—are commonly used to characterize the preeruptive volatile budgets of magmas and to determine magma crystallization pressures and depths from geobarometry (*12*). However, melt inclusions are not always as adept at preserving preeruptive melt volatile contents as might be hoped (*13*–*15*). Experiments and observations of natural melt inclusions suggest that, in some minerals, inclusion H_2_O contents can be modified by diffusive H_2_O exchange between an inclusion and the external melt through the crystal host. This is an important realization, as several common magmatic processes (e.g., degassing due to crystallization or ascent, mixing of magmas, and entrainment of crystals hosting melt inclusions in a different magma) can produce the chemical potential gradient in H_2_O between an inclusion and the external melt that is necessary to drive diffusive exchange.

In the case of mafic magmas, olivine-hosted melt inclusions are the primary target of volatile studies. In these inclusions, the diffusive H_2_O exchange process has been unequivocally verified to occur in both experiments and natural datasets (*14*–*19*). Experiments suggest it can happen rapidly, over timescales relevant to magma eruption (hours to days), and that hydrogen isotopes can record the process (*15*, *17*, *18*). In silicic systems, volatile studies dominantly use quartz-hosted melt inclusions, and experimental studies suggest that diffusive H_2_O exchange is also a concern in these cases (*20*, *21*). However, studies of natural samples are more equivocal: Myers *et al.* (*22*) interpreted scatter in H_2_O contents of early Bishop Tuff quartz-hosted melt inclusions as due to diffusive H_2_O loss during slow ascent and magma stalling. However, Befus *et al.* (*23*)—the only study we know of that has characterized both volatiles and hydrogen isotopes in natural quartz-hosted melt inclusions—found notable variability in both H_2_O and hydrogen isotopes but no clear indication that these variations were caused by diffusive exchange. This disparity highlights a need for more studies of coupled volatile and hydrogen isotope measurements to improve our interpretations of quartz-hosted melt inclusion volatile signatures and further assess whether quartz-hosted inclusions are reliable recorders of preeruptive storage conditions in silicic magmas.

The preferred targets for melt inclusion studies are naturally glassy, nondecrepitated (i.e., unruptured), and mineral inclusion free, as these are most likely to preserve magmatic conditions from the time of entrapment (*24*, *25*). Studies of devitrified (i.e., crystallized) melt inclusions have used laboratory homogenization to reproduce glassy inclusions and allow for quantitative analyses (*26*–*28*). While previous work has suggested that laboratory homogenization likely has a minimal effect on the abundance of dissolved H_2_O in devitrified Bishop Tuff quartz-hosted melt inclusions (*29*), the impacts of homogenization on hydrogen isotope signatures and other dissolved volatiles have not been identified. Devitrified melt inclusions are common, and in some rocks they are ubiquitous (*30*). If laboratory homogenization of quartz-hosted melt inclusions leaves volatiles unaltered relative to what they were at the time of entrapment, then these inclusions can provide vital information about preeruptive magmas that would be otherwise inaccessible.

**Fig. 1.**
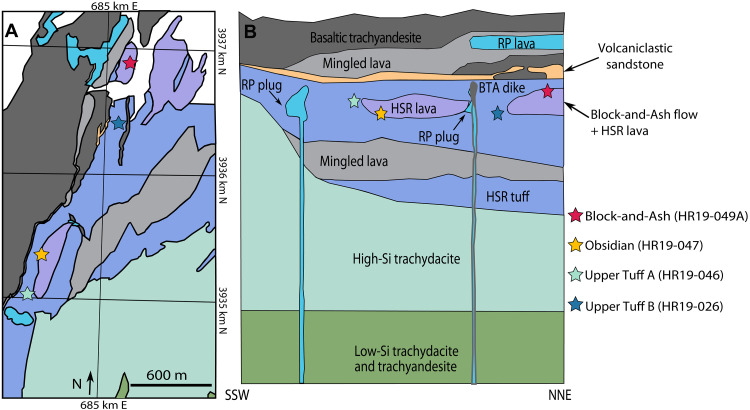
Geologic map and stratigraphic reconstruction. (**A**) Geologic map of Highland Range volcanic sequence and (**B**) reconstruction of volcanic stratigraphy after (*31*, *32*). The location and relative stratigraphic level of each sample are denoted by stars. HSR, high-silica rhyolite; BTA, basaltic trachyandesite; RP, rhyodacite porphyry; SSW, south-southwest; NNE, north-northeast.

Here, we consider these issues as we investigate the hydrogen isotope, trace element, and volatile records of naturally glassy and experimentally homogenized quartz-hosted melt inclusions from a series of explosive and effusive high-silica rhyolite eruptions in the Miocene-age Highland Range volcanic sequence [NV, USA (*31*–*33*)]. We study four rhyolitic units: a block-and-ash flow (Block-and-Ash), an obsidian flow (Obsidian), and two pyroclastic flows (Upper Tuff A and Upper Tuff B; “Tuffs” when discussed in tandem) (Fig. 1). These units represent four eruptive events in the Highland Range volcanic sequence. There is no field evidence [e.g., paleosols and reworked horizons (*31*, *34*, *35*)] of hiatuses between the eruptions, and the Obsidian and Block-and-Ash are enveloped by Tuff deposits equivalent to Upper Tuff A and B. The ages of these eruptions are bracketed by the ~16.3-Ma underlying high-silica trachydacites and the overlying mingled lava at 16.0 Ma (Fig. 1) (*34*), and they have been linked in previous work to a section of the coeval Searchlight pluton that crystallized over ≤90 ka (*36*, *37*). The quartz-hosted melt inclusions from the Tuffs are predominantly glassy and pristine, but those from the Obsidian and Block-and-Ash are devitrified and required homogenization for further compositional characterization.

We use our data for two overarching objectives. We first evaluate the reliability of preeruptive information in naturally glassy and experimentally homogenized quartz-hosted melt inclusions, and we provide, to our knowledge, the first hydrogen isotope measurements in experimentally homogenized quartz-hosted melt inclusions. We then consider the architecture and evolution of the Highland Range high-silica rhyolites. Specifically, we (i) establish the presence and preeruptive distribution of distinct magmas in the crust, (ii) consider how this distribution evolved through the eruptive sequence, and (iii) assess the relationship between dissolved volatiles and eruption explosivity.

Our results demonstrate that quartz-hosted melt inclusions can experience diffusive H_2_O exchange, and it can happen during homogenization. However, they also highlight that with careful sample selection, it is possible to obtain meaningful magmatic signatures from experimentally homogenized inclusions. If primary magmatic hydrogen isotopic compositions are known, we show that we can also correct melt inclusion compositions that have diffusively lost some H_2_O to initial H_2_O concentrations from the time of entrapment. When we consider our reliable melt inclusion compositions from the Highland Range, we observe distinct differences in the volatile, hydrogen isotope, and trace element compositions of the different units. Processes such as degassing and diffusive H_2_O exchange alone cannot explain all these observations. Rather, our results indicate that the magmas that erupted to produce the Obsidian and Block-and-Ash flows were distinct from those that produced the Tuffs, suggesting that a complex magmatic plumbing system was present in the upper crust over the history of these silicic eruptions.

## RESULTS

File S1 contains all melt inclusion data described below. The Supplementary Materials contain all modeling results.

### Sample and melt inclusion descriptions

**Fig. 2.**
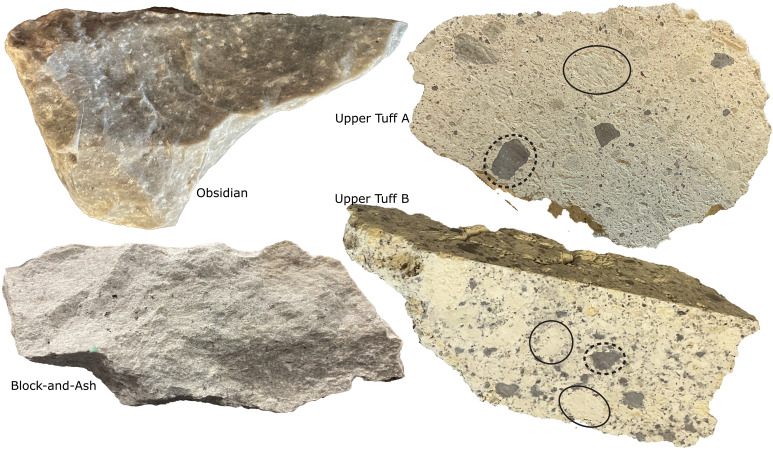
Hand sample images. The rhyolite unit of origin for each hand sample is labelled next to each image. The Block-and-Ash is a single vesiculated block. A pumice clast and a lithic clast are circled on the Upper Tuff B and Upper Tuff A images in solid black and dotted black lines, respectively.

We selected quartz crystals containing melt inclusions from four samples (Obsidian sample HR19-047, Block-and-Ash sample HR19-049a, Upper Tuff A sample HR19-046, and Upper Tuff B sample HR19-026; see Materials and Methods). The Upper Tuff A and Upper Tuff B quartz grains are from small (centimeter-scale) pumice clasts extracted from unwelded pyroclastic flow deposits. The Tuffs contain naturally glassy quartz-hosted melt inclusions. We observed a population of Tuff melt inclusions with vapor bubbles and/or evidence for devitrification/decrepitation (e.g., emanating cracks and/or bubbles and very bubbly glass), and we did not analyze these inclusions. Block-and-Ash quartz grains are from a single, highly vesiculated, glassy block. We extracted Obsidian quartz grains from a nonvesiculated, dark gray, glassy hand sample (Fig. 2).

Large (>25-μm diameter) quartz-hosted melt inclusions from the Obsidian and Block-and-Ash are nearly ubiquitously devitrified. Among all the quartz grains retrieved from these samples, only the smallest quartz-hosted melt inclusions (<20-μm diameter) are glassy, but they contain large, CO_2_-bearing (confirmed by Raman spectroscopy) vapor bubbles and were too small for further analysis. Several of the Obsidian melt inclusions we analyzed contain bubbles and were likely decrepitated. Quartz phenocrysts from all samples discussed here are commonly highly fractured and crosscut by glass-filled cracks. The glass-filled fractures were likely introduced during the eruptive process and are not the result of sample preparation; however, some melt inclusions had small cracks emanating from them that appeared after polishing.

The nearly ubiquitous devitrification of large Block-and-Ash and Obsidian melt inclusions suggests that these units experienced slower posteruptive cooling than the Tuffs. In contrast, the matrix glass of the Block-and-Ash and Obsidian is vitreous. The low H_2_O contents of the degassed matrix melts relative to the melt inclusions may have allowed the former to remain glassy during posteruptive cooling (*38*).

**Fig. 3.**
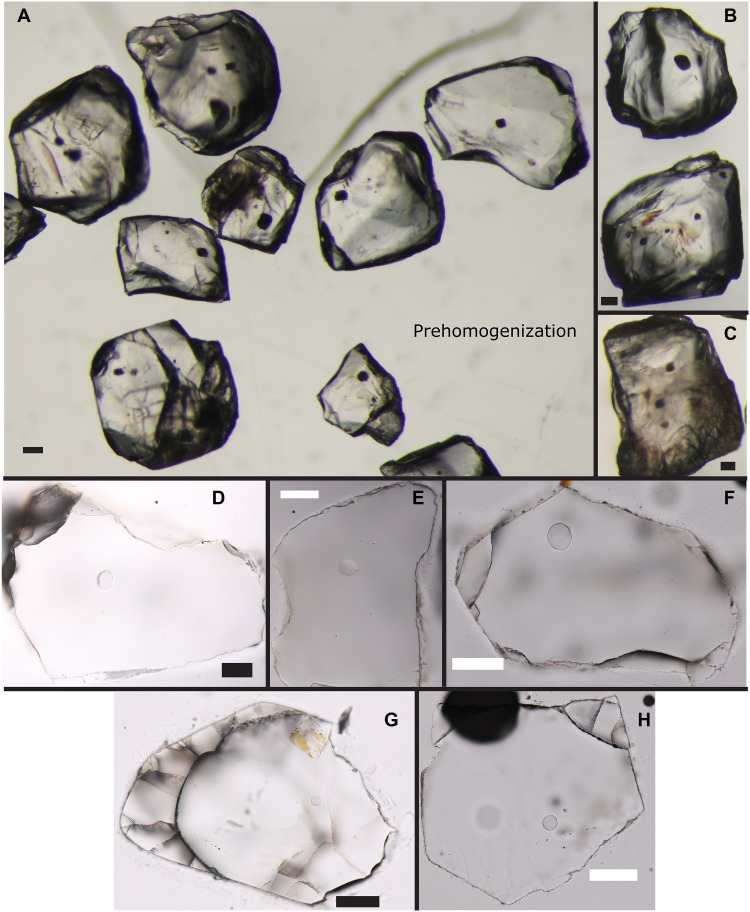
Melt inclusion images. (**A** to **C**) Quartz grains containing devitrified melt inclusions before homogenization experiments. Note the dark colors and rounded to bipyramidal shapes of the melt inclusions. (**D** to **H**) Quartz with glassy melt inclusions after laboratory homogenization. Note the transparent-translucent glass and rounded to irregular inclusion shapes. The scale bar in each image has a width of 100 μm.

Before laboratory homogenization, melt inclusions from the Block-and-Ash and Obsidian appeared dark and generally round to hexagonal bipyramidal in shape. Their dark brown colors, in combination with pervasive quartz fracturing, prevented the identification of vapor bubbles in devitrified melt inclusions before homogenization. After homogenization, melt inclusions are typically colorless with uneven rounded boundaries (Fig. 3). Some homogenized melt inclusions contained a vapor bubble, but we avoided analyzing these inclusions because of the uncertainties associated with reconstructing melt inclusion CO_2_ exsolved in vapor bubbles (*39*). Tuff melt inclusions are colorless to light brown in color and round to hexagonal bipyramidal in shape. Cathodoluminescence maps of representative quartz grains from each sample reveal complex internal zoning patterns (fig. S1). Melt inclusions are entrapped within crystal cores or interiors mantled by rounded growth zones that may be due to quartz resorption. We therefore cannot confidently estimate the relative timing of entrapment of individual melt inclusions.

### Melt inclusion sizes and positions

**Fig. 4.**
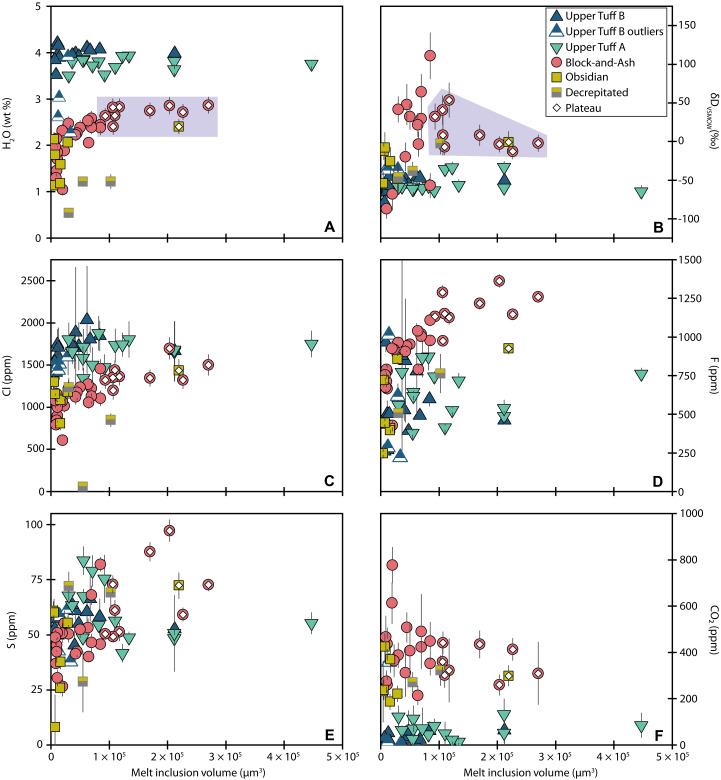
Melt inclusion sizes. Melt inclusion volume versus (**A**) H_2_O, (**B**) δD_VSMOW_, (**C**) Cl, (**D**) F, (**E**) S, and (**F**) CO_2_. The region denoted as the plateau for the Block-and-Ash and Obsidian samples is denoted in purple in (A) and (B). Error bars are 2σ, and those not shown are smaller than the symbol size. The outliers in CO_2_ and F from fig. S5 are not shown here for simplicity.

Melt inclusions from all samples cover a similar range in size (Fig. 4 and file S1). Block-and-Ash (sample 049a) inclusions range from ~7560 to 270,000 μm^3^ in volume and are located 77 to 438 μm away from the nearest host crystal edge (see Materials and Methods for measurement techniques). Obsidian (sample 047) inclusions range from ~577 to 219,000 μm^3^ and are located 56 to 232 μm away from the nearest crystal edge. We refer to the largest melt inclusions from the Block-and-Ash (*n* = 9) and Obsidian (*n* = 1) as the “plateau” melt inclusions (see Volatiles—H_2_O, CO_2_, and vapor saturation pressures section). Block-and-Ash plateau melt inclusions range from ~93,400 to 270,000 μm^3^, and the Obsidian plateau melt inclusion size is ~219,000 μm^3^. Upper Tuff B (sample 026) inclusions range from ~6700 to 212,000 μm^3^ and are located 34 to 165 μm away from the nearest crystal edge. Upper Tuff A (sample 046) inclusions range from ~9780 to 447,000 μm^3^ and are located 52 to 257 μm away from the closest crystal edge (Fig. 4 and figs. S2 and S3).

### Major and trace element compositions

Major element compositions of all measured melt inclusions are high-silica rhyolite (≥75 wt % SiO_2_; file S1). Block-and-Ash and Obsidian inclusions extend to higher SiO_2_ contents than Tuff inclusions, but inclusions in all samples with 76 to 78 wt % SiO_2_ have major element compositions that are similar in all oxides. The smallest melt inclusions from the Block-and-Ash and Obsidian are the most enriched in SiO_2_ and depleted in all other major elements (fig. S4). This is likely a result of quartz dissolution during laboratory homogenization (see Discussion). The Tuff melt inclusion compositions are not enriched in SiO_2_ in any melt inclusion size range.

**Fig. 5.**
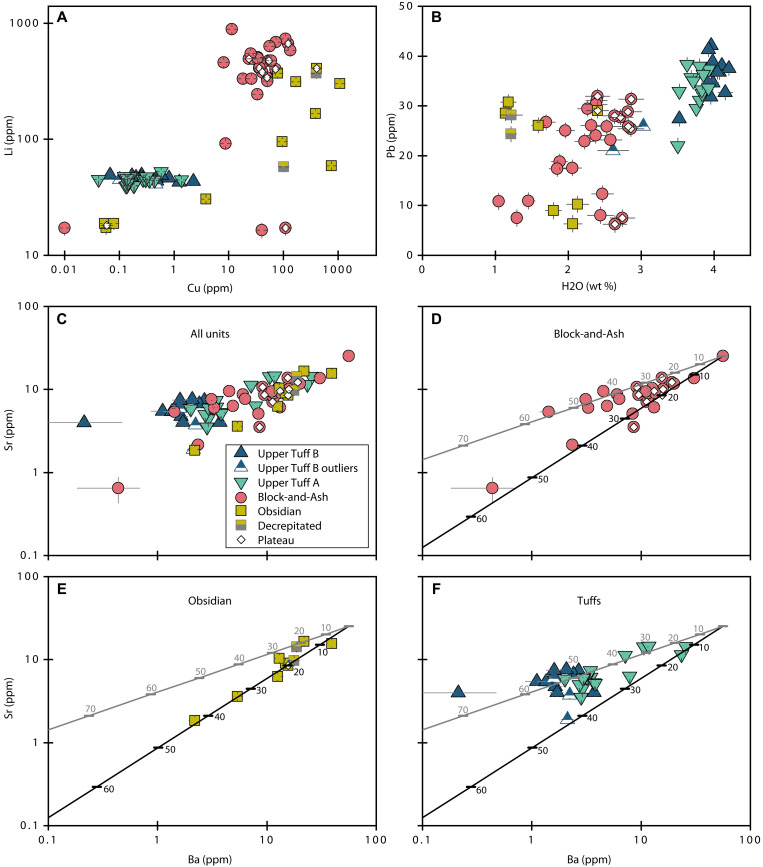
Melt inclusion trace elements. (**A**) Cu versus Li. (**B**) H_2_O versus Pb, two incompatible species in vapor-saturated melts. (**C**) Ba versus Sr, two compatible elements. (**D** to **F**) Fractional crystallization model results for (D) Block-and-Ash, (E) Obsidian, and (F) Tuffs (both samples). Phase assemblage A is shown as a gray line, and phase assemblage B is shown as a black line (see Materials and Methods for details of phase assemblages). The percentage of crystallization is listed along each modeled path in the same color as the path. Note the relatively limited compositional range observed in the Tuff melt inclusions compared to the Block-and-Ash and Obsidian melt inclusions. Error bars are 2σ or may be smaller than symbol sizes.

Trace element differences between the Block-and-Ash and Obsidian melt inclusions and the Tuff melt inclusions are subtle, with some exceptions. The Tuff melt inclusions tend to have higher concentrations of incompatible elements (Rb, Ga, Yb, U, and Pb) and lower abundances of compatible elements (Ba and Sr) compared to the Obsidian and Block-and-Ash melt inclusions (Fig. 5 and file S1). The Obsidian and Block-and-Ash melt inclusions are enriched in Li [up to 886 parts per million (ppm)] and Cu (up to 1081 ppm) relative to the highest measured values of these elements in the Tuff melt inclusions (up to 53 ppm of Li and 5 ppm of Cu; Fig. 5A).

In Ba versus Sr space, the Upper Tuff B melt inclusions contain relatively depleted and uniform concentrations of compatible elements compared to melt inclusions from the other three samples. The Upper Tuff B inclusions contain up to 4 ppm of Ba and 2 to 8 ppm of Sr. Most of the Upper Tuff A inclusions have similar or only slightly higher Ba and Sr contents than Upper Tuff B inclusions. There are also several melt inclusions from Upper Tuff A with Ba and Sr contents that resemble many Block-and-Ash and Obsidian melt inclusion compositions (Fig. 5C). The Block-and-Ash melt inclusions have the greatest variability in Ba and Sr contents of all the samples, ranging from <1 to 56 ppm of Ba and <1 to 25 ppm of Sr, but most Block-and-Ash inclusions contain ≤30 ppm of Ba (25 of 27 measurements) and ≤15 ppm of Sr (28 of 29 measurements). The Obsidian melt inclusions contain 2 to 40 ppm of Ba and <1 to 17 ppm of Sr.

Tuff melt inclusions tend to be more enriched in incompatible Pb than melt inclusions from the Block-and-Ash and Obsidian. Upper Tuff B melt inclusions contain 21 to 42 ppm of Pb (median: 37 ppm of Pb), Upper Tuff A melt inclusions contain 22 to 38 ppm of Pb (median: 34 ppm of Pb), Block-and-Ash melt inclusions contain 6 to 32 ppm of Pb (median: 25 ppm of Pb), and Obsidian melt inclusions contain 6 to 31 ppm of Pb (median: 25 ppm of Pb; Fig. 5B).

### Volatiles—H_2_O, CO_2,_ and vapor saturation pressures

**Fig. 6.**
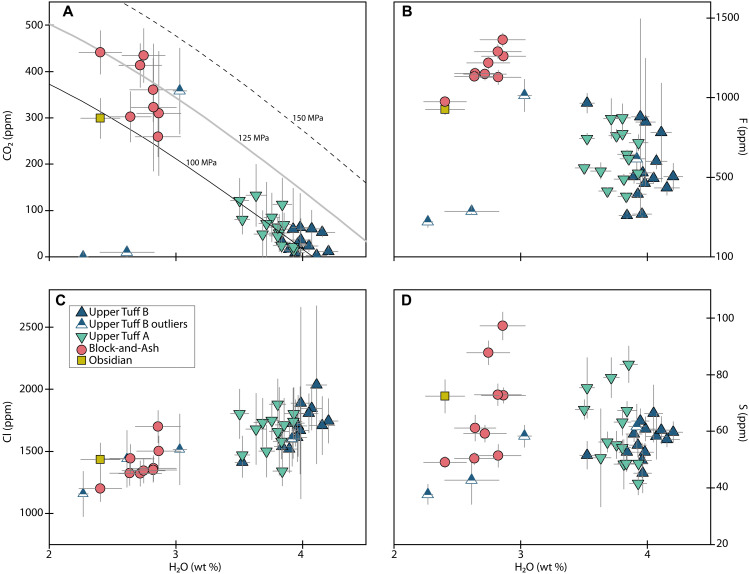
Melt inclusion volatiles. Melt inclusion H_2_O versus (**A**) CO_2_, (**B**) F, (**C**) Cl, and (**D**) S concentrations for plateau melt inclusions only as denoted in Fig. 4. The full melt inclusion suit is shown in fig. S5. The black, gray, and dotted lines on (A) represent calculated 100, 125, and 150 MPa of isobars, respectively. Error bars are 2σ or may be smaller than symbol sizes.

Melt inclusions from the Block-and-Ash contain 213 to 613 ppm of CO_2_ (median: 376 ppm) and 1.0 to 2.9 wt % H_2_O (median: 2.4 wt %; Fig. 6A and fig. S5). We designate two high-CO_2_ inclusions (049a-g14: 9587 ppm and 049a-g23: 777 ppm) as outliers because they lie outside of 1.5× the interquartile range (IQR) of the sample, a commonly applied statistical fence for identifying outliers (see Materials and Methods). Larger melt inclusions from the Block-and-Ash tend to contain higher H_2_O than smaller melt inclusions; we do not observe this relationship in CO_2_ (Fig. 4 and fig. S3). Some of the smallest Block-and-Ash melt inclusions have the highest CO_2_ concentrations, but there is considerable range in small melt inclusion CO_2_ contents. There is no relationship between melt inclusion position in a crystal and either H_2_O or CO_2_ (fig. S2). The largest Block-and-Ash melt inclusions have conspicuously stable, high H_2_O contents (Fig. 4 and fig. S3); we refer to these as plateau inclusions. The median saturation pressure from Block-and-Ash plateau inclusions is 119 ± 24 MPa (2σ).

Melt inclusions from the Obsidian have CO_2_ and H_2_O concentrations ranging from 187 to 425 ppm of CO_2_ (median: 271 ppm) and 0.6 to 2.4 wt % H_2_O (median: 1.4 wt %), respectively (Fig. 6 and fig. S5). The largest Obsidian melt inclusion (047-g46A; the Obsidian plateau inclusion) contains the highest measured H_2_O in this sample and lies on the Block-and-Ash plateau in Fig. 4A. Its CO_2_ concentration is comparable to that of several smaller Obsidian inclusions (Fig. 4 and fig. S3). Similar to the Block-and-Ash, the highest CO_2_ concentrations measured in Obsidian melt inclusions come from small melt inclusions. There is a subtle relationship between position and H_2_O in the Obsidian melt inclusions, where the inclusions farthest from the host crystal edge tend to have higher H_2_O contents (fig. S2). The saturation pressure returned for the single Obsidian plateau inclusion is 109 MPa.

**Fig. 7.**
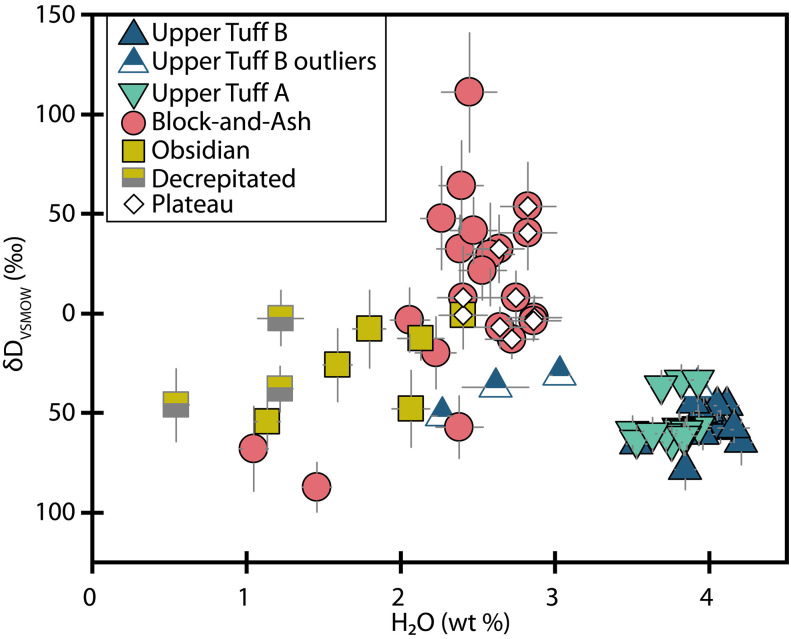
Melt inclusion hydrogen isotopes. Melt inclusion H_2_O versus δD_VSMOW_. The plateau is as denoted in Fig. 4. Error bars are 2σ or may be smaller than symbol sizes.

**Fig. 8.**
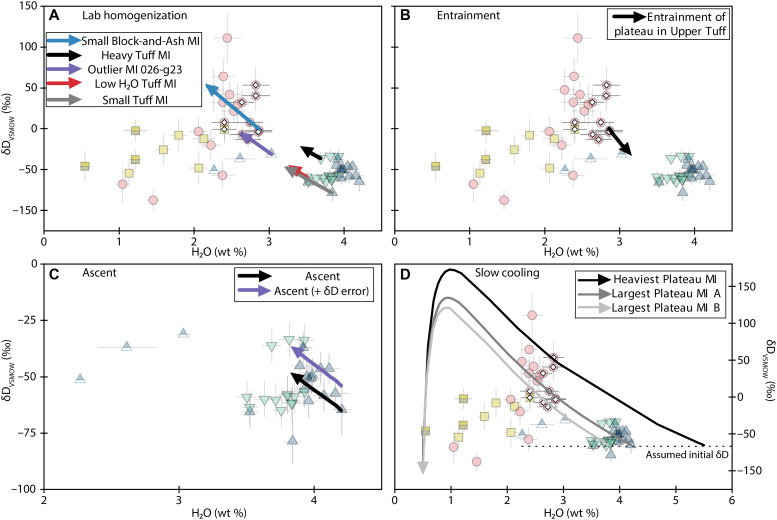
Diffusive H_2_O exchange models. Symbols are the same as in Fig. 4 to 7 but translucent to improve legibility. The symbol at the base of each arrow represents the initial composition, and the tip of each arrow represents the final composition at the end of the model. MI, melt inclusion. In cases where we tested multiple crystal sizes, we plot model results using the smallest crystal size to show the maximum extent of diffusive exchange. The effects of this parameter are small relative to the error on our H_2_O and δD_VSMOW_ measurements. Error bars are 2σ or may be smaller than symbol sizes. See the Supplementary Materials and file S2 for more details on diffusive exchange modeling. (**A**) Diffusive exchange during laboratory homogenization for various initial compositions and melt inclusion geometries. See Materials and Methods for information about homogenization conditions and the Supplementary Materials for information about different initial model conditions. (**B**) Diffusive exchange during entrainment of a plateau melt inclusion within the Upper Tuff magma over 14 hours. This case models the potential origin of Upper Tuff outlier 026-g23. (**C**) Diffusive exchange during eruptive ascent of the Upper Tuff over 8 hours. The black arrow shows diffusive exchange using the measured composition of the highest H_2_O melt inclusion, and the purple arrow shows model results from adding the analytical uncertainty to this melt inclusion’s δD_VSMOW_ measurement. These two end-members encompass most of the Upper Tuff melt inclusion data. (**D**) Diffusive exchange during slow cooling of plateau melt inclusions assuming the initial magmatic δD_VSMOW_ = −65‰, as represented by the dashed line. The different arrows represent models for slow cooling of the largest measured (“Large Plateau MI A and B,” assuming two potential initial H_2_O contents) and the isotopically heaviest (“Heaviest Plateau MI”) melt inclusions.

**Fig. 9.**
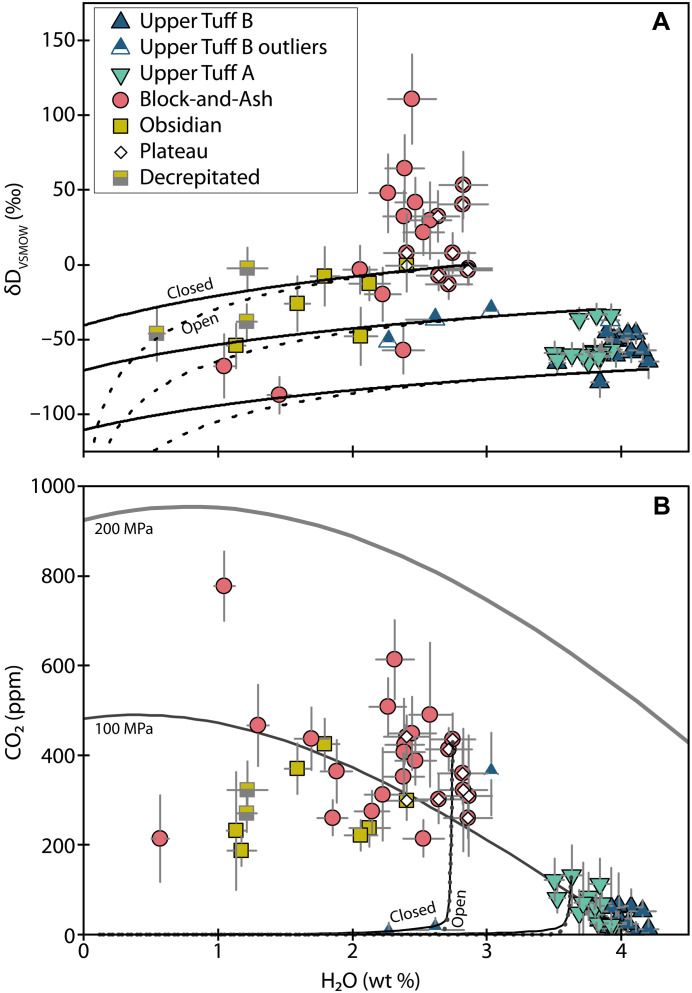
Degassing models. (**A**) H_2_O versus δD_VSMOW_ degassing trends for open- (dotted lines) and closed-system (solid lines) degassing. Model results shown in (A) use two Tuff end-members and a typical plateau composition as initial conditions. (**B**) H_2_O versus CO_2_ degassing models for open- (dotted lines) and closed-system (solid lines) degassing. Model results are shown for the highest CO_2_ melt inclusion from the Block-and-Ash plateau and the Upper Tuff A, respectively. The 100- and 200-MPa isobars are in black and gray, respectively.

CO_2_ in Upper Tuff B melt inclusions varies from <1 ppm up to 63 ppm of CO_2_ (median: 24 ppm) and 3.5 to 4.2 wt % H_2_O (median: 4.0 wt %; Fig. 6 and fig. S5). Two high-CO_2_ and three low-H_2_O inclusions are outliers based on the IQR test (026-g23: 358 ppm of CO_2_, 026-g27: 2093 ppm of CO_2_, 026-g23: 3.0 wt % H_2_O, 026-g29b: 2.3 wt % H_2_O, and 026-g4: 2.6 wt % H_2_O). Three of the smallest melt inclusions analyzed from the Upper Tuff B are relatively depleted in H_2_O and are distinct from the rest of the Upper Tuff B melt inclusions in several other compositional parameters (low Cl, F, S, Pb, and Rb; “Upper Tuff B outliers” in Figs. 4 to 9). We deem these to be outliers and do not interpret their volatile signatures in detail because it is not clear whether they are directly related to the other “main population” Upper Tuff B melt inclusions; we define the main population as all inclusions except these three outliers. Within the main population, there are no strong relationships between melt inclusion size or position and H_2_O or CO_2_ (Fig. 4 and fig. S2). One outlier from the Upper Tuff B (026-g23) is notable because it has H_2_O (3.0 wt %) and CO_2_ (258 ppm of CO_2_) contents that resemble the Block-and-Ash plateau inclusions; this relationship exists for all volatile species (Fig. 6 and fig. S5). The calculated saturation pressure for this outlier inclusion is also slightly deeper (132 MPa) than the rest of the Upper Tuff B inclusions [104 ± 15 MPa (2σ)].

Melt inclusions from the Upper Tuff A plot closely with those from the Upper Tuff B but contain slightly more CO_2_ and less H_2_O, with a range of 14 to 133 ppm of CO_2_ (median: 67 ppm) and 3.5 to 3.9 wt % H_2_O (median: 3.8 wt % H_2_O; Fig. 6 and fig. S5). We observe no strong relationships between melt inclusion size or position and H_2_O, but there is a subtle trend in CO_2_ where some of the smallest melt inclusions have slightly higher CO_2_ concentrations than larger inclusions (Fig. 4 and fig. S2). The median saturation pressure retrieved from the Upper Tuff A melt inclusions is 102 ± 11 MPa (2σ). Block-and-Ash and Obsidian melt inclusions contain less H_2_O and more CO_2_ than Tuff melt inclusions except for the Upper Tuff B outliers (Fig. 6 and fig. S5).

### Volatiles—Cl, F, and S

Block-and-Ash and Obsidian plateau melt inclusions have similar or slightly lower Cl concentrations than those from the Tuffs, and Cl is positively correlated with H_2_O across the sample populations (Fig. 6C). The Block-and-Ash plateau melt inclusion compositions extend to higher F and S contents than in the Tuff melt inclusions (Fig. 6 and fig. S5). The Obsidian melt inclusion suite (including smaller, nonplateau inclusions) has one high F outlier (047-g52; 21700 ppm of F), and the Block-and-Ash melt inclusion suite has one low F outlier (049a-g18: 168 ppm of F; file S1).

Melt inclusions from the Block-and-Ash and Obsidian have higher measured concentrations of Cl, F, and, to a lesser extent, S, at larger melt inclusion sizes, but we observe no correlations with melt inclusion position (fig. S2). Plateau melt inclusions are typically more enriched in volatiles than nonplateau inclusions (Fig. 4 and fig. S5). We do not observe any relationships between melt inclusion size or position with volatile contents in either Tuff sample (Fig. 4 and fig. S2).

### Hydrogen isotopes (δD_VSMOW_)

Block-and-Ash and Obsidian melt inclusion δD_VSMOW_ values range from −87 to +111 and −54 to 0‰ (per mil), respectively (Fig. 7), where

$${\delta D}_{\text{Vienna Standard Mean Ocean Water}\left(\text{VSMOW}\right)}=\left(\frac{D/H_{\text{unknown}}}{D/H_{\text{VSMOW}}}-1\right)\times 1000$$ (1)

Small inclusions (<0.93 × 10^5^ μm^3^) from the Block-and-Ash and Obsidian display more isotopic variation than larger (i.e., plateau) inclusions (Fig. 4B and fig. S3). Many of the small Block-and-Ash inclusions are also isotopically heavier than larger inclusions from this sample. Together, the suite of Block-and-Ash inclusions forms a negative correlation between H_2_O concentration and δD_VSMOW_, but four melt inclusions with more negative δD_VSMOW_ than the plateau do not fall on this trend (Fig. 7). Most Obsidian melt inclusion δD_VSMOW_ values are isotopically lighter than the Block-and-Ash inclusions, and relationships between Obsidian melt inclusion δD_VSMOW_, H_2_O, and sizes are less clear (Figs. 4 and 7 and fig. S3). Relationships between melt inclusion δD_VSMOW_ and location relative to the crystal edge are not evident in either of these samples (fig. S2).

Hydrogen isotope values measured in the Tuff inclusions are less variable and isotopically lighter than the Block-and-Ash and Obsidian inclusions (Fig. 7 and file S1). The Upper Tuff B δD_VSMOW_ values are between −79 and −31‰, and the Upper Tuff A inclusions are between −65 and −33‰ (Fig. 7). We do not observe any strong relationships between melt inclusion size and δD_VSMOW_ for either sample (Fig. 4 and fig. S3). All but two melt inclusions in the main population of Upper Tuff B inclusions (13 of 15 measurements) show a negative correlation between H_2_O and δD_VSMOW_ (Fig. 7).

## DISCUSSION

Previous studies suggest that melt inclusion compositions can experience postentrapment modification via diffusive exchange (*15*, *20*); thus, it is especially important to carefully consider whether the compositions of our natural melt inclusions were modified by diffusive exchange before using them to make interpretations about magmatic conditions. The vulnerability of a melt inclusion to diffusive loss or gain of an element or molecule is primarily related to the size and position of the melt inclusion within its host crystal, the abundance of the element or molecule in the host crystal, and the ability for that element or molecule to diffuse through the host crystal (*13*, *40*). In principle, smaller melt inclusions and those closer to the rim of a crystal are most susceptible to compositional changes from diffusive exchange; their higher surface area to volume ratios allow for a larger proportion of melt inclusion contents to diffusively equilibrate with the external environment over a given period of time (*13*).

As we discuss in more detail, we find that volatiles, excluding CO_2_, may be sensitive to diffusive exchange in quartz, but trace elements are not as susceptible to it. We demonstrate that relationships between melt inclusion composition and geometry are especially useful for identifying diffusive exchange of elements, particularly those that cannot be traced by isotopic fractionation (*41*), although coexisting melt inclusions in a crystal may modulate the composition (particularly H_2_O) of neighboring inclusions and lead to complex diffusive exchange signatures in natural samples (*42*). We show that large (>~60-μm average diameter or >~9.3 × 10^4^ μm^3^) melt inclusions can retain natural volatile signatures and are the least affected by assimilation of host quartz during experimental homogenization, but smaller inclusions may be rendered unusable (except in CO_2_). We also demonstrate that if initial melt inclusion H_2_O or δD_VSMOW_ before diffusive exchange can be determined, then many melt inclusions with partially reequilibrated compositions can still be corrected to original values.

Our results ultimately illustrate that it is crucial to select large melt inclusions for volatile characterization, whether they are naturally glassy or homogenized in the laboratory. At the same time, they highlight the importance of studying a wide array of melt inclusion sizes to effectively identify evidence for diffusive exchange in quartz-hosted melt inclusions. Applying the results of our most reliable melt inclusions, we find that the Highland Range rhyolites provide a record of a complex magmatic system composed of multiple discrete magmas in space and/or time.

### Diffusive exchange and modification of melt inclusion major and trace elements

Experimental studies indicate that some elements, particularly univalent cations (Li, Na, Ag, and Cu), can diffuse rapidly in quartz (*41*, *43*–*46*). This suggests that laboratory homogenization might affect the natural trace element signature of melt inclusions. To consider this, we leverage the rapid diffusion of Li in quartz (*21*).

Since there is no external source of Li in our experimental setup, homogenization could have induced diffusive loss of Li. However, our calculations indicate that ≤~10% of the Li in the inclusions would have been lost during our experiments (Supplementary Materials), which is near the limits of uncertainty on our Li measurements (~3 to 10%). We argue that the other trace elements we consider (Ba, Sr, Cu, and Pb) have also not been extensively modified by diffusion during laboratory homogenization, because these species diffuse more slowly than Li in quartz (*47*). This conclusion is supported by the lack of correlations between melt inclusion volume and trace element concentration in our datasets (fig. S4).

Slow posteruptive cooling of the Block-and-Ash and Obsidian could have also led to diffusive modification of trace elements in melt inclusions, particularly Cu and Li. Experiments suggest that Li is fluid mobile in silicic melts only at very low pressures [<50 MPa (*46*)], and posteruptive surface degassing can gradually deplete the residual melt in Li during slow cooling. This could lead to diffusive loss of Li from inclusions. However, this process competes with the effect of crystallization during cooling, which can enrich the residual melt in incompatible Li (*48*, *49*). In addition, experiments suggest that Li^+^ can diffuse into quartz to charge balance H^+^ diffusing out (*21*, *50*). So, if a melt inclusion undergoes diffusive H_2_O loss during slow cooling, it could diffusively gain Li. In contrast, experiments suggest that Cu is fluid mobile at greater depths and will preferentially enter the fluid phase once fluid saturation is reached (*48*, *51*). The strong fluid partitioning behavior of Cu relative to Li during pre-, syn-, and posteruptive conditions means that Cu is unlikely to be enriched in residual glasses of fluid-saturated magmas that experience slow cooling. Consequently, there is no apparent driver for natural Cu gain in the melt inclusions. In all cases, small inclusions would be particularly susceptible to diffusive equilibration, but we observe no systematic evidence of trace element depletion (or enrichment) in small melt inclusions (fig. S4). Thus, we conclude that the observed trace element variations in our samples are not the result of postentrapment diffusive exchange at emplacement, with the possible exception of Li.

The unreasonably high measured SiO_2_ concentrations (>79 wt % SiO_2_) and amorphous rounded shapes and ragged edges (Fig. 3) of the homogenized Obsidian and Block-and-Ash melt inclusions suggest that some of the host quartz melted during laboratory homogenization (file S1) (*27*). This would dilute the trace element concentrations in the melt inclusions, and we estimate that host quartz dissolution would have diluted all compositional components other than SiO_2_ (and any trace elements the quartz contains, e.g., Ti and Al) by ≤~33% (the Supplementary Materials). Assuming the dissolution rate of the host quartz during homogenization is independent of melt inclusion size, smaller melt inclusions should incorporate proportionally more SiO_2_ than larger inclusions.

We do see evidence for quartz resorption as a function of melt inclusion size in the major element, but not trace element, compositions of the homogenized melt inclusions. Relatively small, homogenized melt inclusions tend to have comparable, and sometimes even higher, trace element concentrations relative to the largest homogenized inclusions (fig. S4). We suggest that the trace element concentrations of the largest homogenized (i.e., plateau) melt inclusions should be the most reliable, but we do not observe differences in trace element compositions between the populations of small and large homogenized melt inclusions. Thus, we do not anticipate that the dilution effect meaningfully affects interpretations of our melt inclusion trace element data. Future studies of devitrified melt inclusions could mitigate compositional dilution if the laboratory homogenization conditions prevent dissolution of the quartz host. For example, Skirius *et al.* (*29*) found no compositional differences between devitrified quartz-hosted melt inclusions homogenized at ~800° to 900°C for ~20 hours and naturally glassy quartz-hosted melt inclusions from the Bishop Tuff. Their experimental conditions may be more suitable for avoiding host quartz dissolution.

In the naturally glassy Tuff samples, there are no clear correlations between melt inclusion major or trace element compositions and inclusion size or position (Fig. 4 and fig. S2). This supports the interpretation that the major and trace element compositions of the these melt inclusions are also not modified by diffusive exchange or host quartz resorption.

Last, it is worth considering whether the melt inclusions represent entrapped boundary layers. If so, then their measured major and trace element compositions may not be representative of magmatic conditions. If rapid quartz crystallization outpaces diffusive equilibration in the melt, then compositional components other than SiO_2_ could become enriched in local regions immediately surrounding quartz crystals. Melt inclusions that sample such a boundary layer could thus be enriched in volatile, major, and trace elements and fail to reflect the “global” melt composition at the time of entrapment (*52*). Small melt inclusions should be especially prone to the effects of such boundary layer entrapment (*53*). Given that we do not observe consistent correlations between trace element concentrations and melt inclusion sizes in any of our samples—the compositional variability in small melt inclusions mirrors that of larger melt inclusions—we argue that there is little trace element evidence for boundary layer entrapment in our melt inclusions.

### Evidence from trace elements for multiple discrete magmas in the Highland Range

Previous studies of the Highland Range demonstrate that the whole-rock major element compositions of the units we studied are near-invariant high-silica rhyolites (file S3) (*31*, *32*), and our major element analyses of the melt inclusion glasses are consistent with this classification (file S1). The major element compositions of the units overlap considerably, but their trace element compositions are distinctive. If diffusive exchange did not modify the trace element signatures of the melt inclusions, then our trace element measurements suggest that the Highland Range rhyolite eruptions tapped magma reservoirs that were discrete in space and/or time.

At face value, the melt inclusion Ba and Sr contents from the different eruptive units appear as if they could be related by progressive fractional crystallization of a common Ba- and Sr-enriched parent magma. The melt inclusions with the highest concentrations of compatible Ba and Sr, and therefore presumably the least-fractionated compositions, come from the Block-and-Ash sample. However, given the low crystal contents of the rhyolites (<10 vol %), we find that fractional crystallization of the most compatible element–enriched Block-and-Ash melt inclusion composition cannot explain either the observed intersample trace element variability or the variability within the Block-and-Ash sample itself (Fig. 5).

The model of progressive crystallization of a single common parent magma is also at odds with the stratigraphy and crystal contents of the rhyolite units. The relatively compatible element–enriched and crystal-poor Block-and-Ash flow erupted after the Obsidian and the Tuffs (*31*). Thus, a more stratigraphically appropriate starting composition for the fractional crystallization model should be from the Obsidian, which erupted first. However, crystallization of the melts from any of the earlier-erupted rhyolites would drive melt trace element concentrations away from those measured in the Block-and-Ash, and the Block-and-Ash does not contain more crystals than the other units. Thus, we conclude that the trace element variability observed through the sequence of Highland Range eruptions cannot be the result of progressive fractional crystallization of a single parental rhyolite magma.

Melt inclusions from the Obsidian and Block-and-Ash are also enriched in Li and Cu relative to the Tuff inclusions (Fig. 5A). We concluded above that these elements were not substantially affected by diffusive exchange during laboratory homogenization, although homogenization could have dissolved Li- and Cu-bearing vapor bubbles and/or mineral inclusions into the Block-and-Ash and Obsidian inclusions. However, we did not observe any secondary phases in the Tuff melt inclusions that could be unmeasured reservoirs for magmatic Cu and Li. As also noted, slow cooling of the Block-and-Ash and Obsidian likely did not affect Cu, but it might have induced diffusive Li gain. However, we see no relationships in Li contents and inclusion size to suggest this. Hence, we suggest that the difference in Cu, and possibly Li, concentrations between the samples reflect original magmatic signatures.

The high degree of intrasample melt inclusion trace element variability, the results of our fractional crystallization models, and the disparity in Cu (and possibly Li) between the samples together suggest that melt compositional heterogeneity during quartz growth and melt inclusion entrapment was pervasive in the Highland Range magmatic system (Fig. 5). Previous studies suggest that the silicic melts that produced the Highland Range eruptions were extracted from a crystal-rich mush at depth [i.e., the Searchlight pluton (*32*, *33*, *54*)] and stored at shallower depths for some time before eruption (*32*, *33*). In this context, we conclude that the melt inclusions from the Highland Range rhyolites likely record the trace element compositions of batches of melt extracted from a shared but locally heterogeneous mushy parent body. Melts extracted from local regions of high and low crystal contents in a common parental mush could have highly variable trace element signatures. For example, the depletion of Sr and Ba in the Upper Tuff B melt inclusions relative to those from the Block-and-Ash and Obsidian suggests that a more extensive history of crystallization is recorded in this sample (Fig. 5). We suggest that the mush region(s) from which the Upper Tuff B magma was initially extracted may have been more crystal rich than those that produced the other units.

Together, our trace element data point toward a model for the Highland Range magmatic system that includes multiple distinct preeruptive reservoirs of rhyolitic magma. From these data, we can distinguish at least two compositional reservoirs; melt inclusions from both Tuff samples are different from the Obsidian and Block-and-Ash in trace elements, but we cannot differentiate Upper Tuff A from Upper Tuff B or the Obsidian from the Block-and-Ash, respectively. These reservoirs may have been discrete in space, suggesting a geometrically complex magmatic architecture, or discrete in time, which would indicate the presence of a longer-lived magma reservoir that emptied and refilled several times. They could also be discrete in both space and time, which would also suggest a complex and dynamic subsurface magmatic architecture.

### Diffusive exchange and modification of melt inclusion H_2_O

If a population of melt inclusions has experienced diffusive H_2_O loss, we expect to observe a negative correlation between H_2_O and δD_VSMOW_, where the lowest H_2_O melt inclusions are also the isotopically heaviest (*55*). Given the importance of melt inclusion volume and location for diffusive exchange, we also expect to see a relationship between the H_2_O and δD_VSMOW_ of a melt inclusion and its volume and position in a crystal (*15*, *18*).

Within this framework, we observe compelling evidence for diffusive H_2_O exchange in the homogenized melt inclusions. First, we see a pronounced decrease in H_2_O with increasing δD_VSMOW_ in the Block-and-Ash dataset (Fig. 7). We also see that the nine largest inclusions from the Block-and-Ash and the largest inclusion from the Obsidian show elevated and consistent H_2_O concentrations and stable δD_VSMOW_ values. In contrast, the smaller inclusions in these samples show variations as a function of size (Fig. 4 and fig. S3).

We rule out the possibility that accidental analysis of the host quartz, due to large analytical spot volumes or errant spot placement, has affected the H_2_O and δD_VSMOW_ measurements because some of the smallest melt inclusions have high concentrations of CO_2_. We would expect CO_2_ to be systematically low in small inclusions if we analyzed substantial host quartz during our volatile analyses (Fig. 4). We also rule out effects of quartz dissolution during homogenization on δD_VSMOW_ because bulk concentrations of H in quartz are very low. Similarly, the variations in H_2_O with size are greater than would be expected from the effects of dilution from quartz dissolution (i.e., >33% variation). Last, we rule out the possibility that boundary layer entrapment during isobaric crystallization has affected the melt inclusion H_2_O and δD_VSMOW_ values in any of our datasets because the rapid diffusivity of H^+^ (and D^+^) in rhyolitic melts at 700° to 750°C (~10^−10^ m^2^/s) would lead to relatively fast equilibration of H_2_O-δD_VSMOW_ (*56*).

Given these considerations, the relationships we observe between H_2_O, δD_VSMOW_, and melt inclusion size in the Block-and-Ash suggest that diffusive exchange of H_2_O did occur from the melt inclusions in our experimentally homogenized samples. Because we conducted the experiments in an H_2_O-free environment, any diffusive exchange due to homogenization would have caused loss, rather than gain, of H_2_O. Our models suggest that laboratory homogenization of small (10-μm radius) melt inclusions with initial plateau-like compositions (2.9 wt % H_2_O; δD_VSMOW_ = 0‰) could reproduce the isotopically heaviest Block-and-Ash melt inclusion compositions (file S2), but they negate the possibility that the Block-and-Ash inclusions originally had compositions akin to the Tuff inclusions before homogenization; after homogenization, Tuff melt inclusions would retain more H_2_O than measured in the plateau (Fig. 8A).

Despite the evidence for diffusive exchange in the small homogenized inclusions, the stability of the H_2_O measurements from the largest ones suggests that the plateau inclusions may retain original H_2_O signatures. The ~60‰ variation in plateau δD_VSMOW_ is also associated with a small range in H_2_O (~0.4 wt %; Fig. 7). If the range in δD_VSMOW_ in the plateau melt inclusions reflected diffusive loss from a single initial H_2_O-δD_VSMOW_ composition, then a larger range (~0.8 wt %) in H_2_O than we observe should accompany the isotopic variability. If the plateau δD_VSMOW_ values are unmodified, then this would suggest that the melt entrapped by the plateau melt inclusions was isotopically heterogeneous in space and/or time.

The slow posteruptive cooling implied by the devitrification of most melt inclusions from the Block-and-Ash and Obsidian could have induced diffusive H_2_O loss from the relatively hydrous melt inclusions to the degassed matrix material. If so, then our homogenized melt inclusions may preserve a natural signature, but the values may not represent original magmatic values. In this case, we can consider the possibility that the naturally glassy Tuff inclusions preserve the original δD_VSMOW_ value of the Highland Range rhyolites. Under this assumption, we can use a similar approach to Walowski *et al.* (*57*) and leverage δD_VSMOW_ in the Tuff inclusions to reconstruct the original magmatic H_2_O value of the homogenized inclusions that follow diffusive exchange curves (i.e., Block-and-Ash melt inclusions that form a negative array in H_2_O-δD_VSMOW_).

The most H_2_O-rich Tuff melt inclusion has a δD_VSMOW_ value of ~−65‰. Assuming this was the original value of the Block-and-Ash and Obsidian melt inclusions, H_2_O loss during slow cooling could have produced the range in measured H_2_O-δD_VSMOW_ if the initial melt contained between 3.8 and 5.5 wt % H_2_O (Supplementary Materials; Fig. 8D). Under these assumptions, melt inclusions that do not follow diffusive exchange curves (i.e., most of the Obsidian melt inclusions and several of the Block-and-Ash melt inclusions with low H_2_O and light δD_VSMOW_ values) may have more fully equilibrated with the degassed external melt during cooling, and their initial H_2_O contents cannot be reliably reconstructed by this approach.

This approach could be a powerful tool for reconstructing original magmatic H_2_O from homogenized quartz-hosted melt inclusions. However, it requires additional information and/or assumptions about the primary magmatic δD_VSMOW_ values or H_2_O concentrations. Befus *et al.* (*23*) provide the only other study, to our knowledge, of H_2_O-δD_VSMOW_ values from undegassed rhyolitic glass inclusions. They determined that quartz-hosted melt inclusions from the Bishop Tuff, with H_2_O contents around 4.2 wt % H_2_O, have primary δD_VSMOW_ values between −40 and −60‰. Our δD_VSMOW_ values from the Tuff inclusions have a similar range, particularly those from Upper Tuff A, which show no compelling evidence for diffusive H_2_O loss (δD_VSMOW_ = −30 to −65‰). This suggests that primary rhyolitic δD_VSMOW_ values can be heterogeneous, and isotopic heterogeneity in magmas can be recorded by quartz-hosted melt inclusions, but the similitude of our results may also suggest that rhyolitic magmas typically have δD_VSMOW_ values in this range. However, our combined studies represent just two datasets on rhyolitic systems. In contrast, Befus *et al.* (*23*) provide a global compilation of basaltic glass inclusion δD values from 10 studies, and δD_VSMOW_ values range substantially from −200‰ up to +50‰. Given the lack of comparable data for rhyolites, we emphasize the need for more studies that report H_2_O-δD_VSMOW_ from rhyolitic melt inclusions to understand the controls on primary rhyolitic H_2_O-δD values. As it stands, our ability to make confident assumptions about primary rhyolitic δD_VSMOW_ signatures before degassing or diffusive exchange across magmatic systems or eruptive units is limited.

Evidently, we cannot unambiguously determine whether the plateau melt inclusion H_2_O and δD_VSMOW_ compositions in the Block-and-Ash and Obsidian samples were modified by diffusive loss during slow posteruptive cooling or whether they preserve primary magmatic values. However, we argue that homogenized quartz-hosted melt inclusions can still be useful for identifying magmatic conditions. Specifically, we suggest that identification of a plateau in H_2_O values provides the most reliable method for assessing whether primary magmatic values are preserved. If primary magmatic values are modified but initial H_2_O or δD_VSMOW_ can be reliably estimated, then plateau inclusions can also be used to reconstruct primary values of these parameters.

In the case of the naturally glassy samples, some melt inclusions from the unwelded Upper Tuff B also show evidence for diffusive exchange, albeit the case is weaker than in the Obsidian and Block-and-Ash. The main population of the Upper Tuff B melt inclusions do not display a consistent relationship between melt inclusion volume and H_2_O or δD_VSMOW_ (Fig. 4), but most melt inclusions do show a negative correlation between H_2_O and δD_VSMOW_ (Fig. 7) consistent with diffusive H_2_O loss. Unlike the Obsidian and Block-and-Ash samples, we see no evidence for slow posteruptive cooling of the Tuffs (*58*), but degassing of the external melt during ascent could induce diffusive H_2_O loss. In the Upper Tuff A dataset, we do not observe compositional or geometric relationships suggestive of diffusive exchange, making this the only eruptive unit in this study that shows no compelling evidence for postentrapment diffusive exchange of melt inclusion H_2_O.

Last, several of the melt inclusions from the Obsidian fall off the trend in melt inclusion volume versus H_2_O (Fig. 4), suggesting that these inclusions are decrepitated. Decrepitation results in bulk loss of melt mass and volatiles from the inclusion, which would not cause isotope fractionation. The two largest Obsidian melt inclusions—one with conspicuously low H_2_O, Cl, and F contents—are from the same grain and have the same δD_VSMOW_ (~0 ‰; Fig. 4), supporting our conclusion that the one with low volatile contents is decrepitated. In this case, while the volatile signature of this relatively large inclusion is unreliable, we argue that the δD_VSMOW_ value is trustworthy.

We conclude that large quartz-hosted melt inclusions can retain key information about magmatic H_2_O and δD_VSMOW_. In many volcanic deposits, melt inclusions are ubiquitously devitrified, and slow, posteruptive cooling could cause diffusive modification of melt inclusion H_2_O and δD_VSMOW_ (*59*). However, we find that laboratory homogenization, even at the high temperatures we used (~1000°C), produces limited diffusive modifications of melt inclusion H_2_O and δD_VSMOW_ in large (≥~60-μm average diameter or >9.3 × 10^4^ μm^3^) melt inclusions. Thus, even if slow cooling has compromised melt inclusion H_2_O or δD_VSMOW_ values, compositions of large, homogenized inclusions can be used to reconstruct primary magmatic values, if primary values of either of these parameters can be constrained independently.

We emphasize that our reconstruction approach would likely be useful for units that show little evidence for extended cooling timescales, but slower cooled deposits may experience too much diffusive modification during posteruptive cooling to retain meaningful natural information, even in very large melt inclusions. Our Tuff samples are both unwelded, and the groundmass materials of the Block-and-Ash and Obsidian are glassy and microlite free, suggesting relatively rapid posteruptive cooling. This differs from welded tuffs, whose cooling rates can be many orders of magnitude slower than for unwelded tuffs (*58*), or volcanic units with extensive groundmass crystallization. Regardless, our results, consistent with conclusions for plutonic rocks (*30*), demonstrate that devitrified quartz-hosted melt inclusions from rhyolites can still be useful for understanding preeruptive magmatic volatiles and geochemistry in many silicic systems.

### Diffusive exchange and modification of melt inclusion Cl, F, S, and CO_2_

To our knowledge, evidence for diffusive loss of Cl, F, and S from melt inclusions has not been previously documented in quartz, but experiments by Portnyagin *et al.* (*60*) indicate that diffusive exchange of F can occur in olivine. The relationships we observe between melt inclusion size and concentrations of Cl, F, and, to a lesser extent, S, in the Block-and-Ash and Obsidian melt inclusions suggest that these elements can also be affected by diffusive exchange through the host quartz (Fig. 4 and fig. S3). Specifically, nonplateau Block-and-Ash and Obsidian melt inclusions have progressively depleted volatile concentrations, except CO_2_, as melt inclusion volume decreases (Fig. 4). While dilution from the host quartz may contribute to this, it is not sufficient to explain the large variations in these volatiles or the degree of enrichment in CO_2_ at small sizes (Supplementary Materials). Entrapment of boundary layer melts could lead to CO_2_-enriched compositions (*61*), but several small Block-and-Ash and Obsidian melt inclusions have similar or lower CO_2_ relative to the large melt inclusions. In combination with the lack of other trace element evidence, we do not interpret the melt inclusions in this study as entrapped boundary layer melts.

Establishing the specific mechanism(s) by which Cl, F, and S diffuse through β-quartz in magmatic settings is beyond the scope of this study. However, the channel between oxygen atoms along the *c* axis in the β-quartz structure (radius ~ 1 Å) enables rapid diffusion of monovalent ions, such as Li^+^ (0.76 Å), Cu^+^ (0.77 Å), and Na^+^ [1.02 Å (*41*, *44*, *45*, *62*–*64*)]. The larger ionic radii of Cl^−^ (1.81 Å), F^−^ (1.33 Å), and S^2−^ [1.84 Å (*64*)] suggest that these species are unlikely to diffuse rapidly through the quartz lattice, but K^+^ (1.38 Å) is also able to diffuse through quartz despite its large size (*46*). This suggests that the size of Cl, F, and S may not be prohibitive for diffusive exchange through quartz. Defects in the quartz structure may also provide larger diffusion pathways for these volatile elements to diffuse through (*65*). We conclude that the compositions of quartz-hosted melt inclusions can be modified by diffusive exchange of Cl, F, and potentially S, but we do not see evidence of CO_2_ exchange.

### Evidence from volatiles and hydrogen isotopes for multiple magmas in the Highland Range

We assume that the diffusive volatile loss signatures we observe in the smallest Block-and-Ash and Obsidian melt inclusions are primarily the result of our homogenization experiments. However, natural processes, such as preeruptive ascent and slow cooling upon emplacement at the surface, could also be the cause and/or have contributed to the diffusive loss signatures. Considering that large melt inclusions from all samples retain consistent volatile concentrations (Fig. 4), the process(es) responsible for the observed diffusive exchange in the small melt inclusions must have occurred either at temperatures low enough or over timescales short enough to leave the plateau inclusions relatively unaffected. Consequently, as noted in the previous section, regardless of which process caused the diffusive loss in small Block-and-Ash and Obsidian inclusions, we can still retrieve meaningful information about volatiles and hydrogen isotope signatures from the plateau inclusions in these samples.

Although the range in reconstructed H_2_O contents from the Block-and-Ash and Obsidian plateau inclusions overlaps with measured Tuff melt inclusion H_2_O, the relative enrichment of CO_2_, F, and trace elements (Ba, Sr, Li, and Cu) in all the Block-and-Ash and Obsidian melt inclusions cannot be explained by diffusive exchange during slow posteruptive cooling. Mass balance calculations show that the CO_2_ content of an Upper Tuff A melt inclusion would increase by only ~4% if the melt inclusion dehydrated entirely through diffusive loss, which is within error of our CO_2_ measurements in this sample (Supplementary Materials). This suggests that the differences we observe between our samples, which are outside of error, reflect distinct magmatic compositions.

It is also unlikely that the differences in H_2_O and CO_2_ between the samples are due to a CO_2_-rich fluid flushing through the system [i.e., “CO_2_ flushing” (*8*)]. Isobaric CO_2_ flushing of a fluid-saturated magma is expected to increase CO_2_ and decrease H_2_O dissolved in the melt along the isobar and drive crystallization (*8*). The observed enrichment in H_2_O and depletion of CO_2_ in the transition from Upper Tuff A to Upper Tuff B is the opposite of this expectation. Similarly, the low crystal contents of the Block-and-Ash and Obsidian are inconsistent with crystallization caused by CO_2_ flushing—the highest CO_2_ melt inclusions come from rocks with the lowest crystal contents. It is also unclear whether CO_2_ flushing can effectively change dissolved volatile compositions in high-silica rhyolites over timescales relevant to preeruptive storage. Experimental investigations into CO_2_ flushing of rhyolitic melts have concluded that the presence of a CO_2_ fluid will rapidly drive magmatic H_2_O down, but diffusion of CO_2_ is sluggish enough that a proportional increase of CO_2_ may not occur; rhyolitic melts remained CO_2_ undersaturated in CO_2_ fluid flushing experiments lasting up to 30 days (*66**,* *67*).

Thus, regardless of how we interpret the plateau H_2_O data—whether they are primary magmatic values or require reconstruction—we see evidence for discrete magmas in the volatile data, much as we did in the trace elements.

Further evidence for this conclusion might also be found in fluid saturation pressures from the melt inclusions, if slow cooling did modify the Block-and-Ash and Obsidian melt inclusions. Specifically, reconstructed H_2_O and elevated CO_2_ contents of Block-and-Ash and Obsidian plateau melt inclusions imply much deeper preeruptive storage of these magmas (~200 to 250 MPa) than those of the Tuffs (~100 MPa); estimates from unreconstructed plateau H_2_O values (~100 to 150 MPa) are more similar to those from the Tuff inclusions. Future detailed geobarometry work on the Highland Range rhyolites and more studies reporting primary rhyolitic δD_VSMOW_ signatures from different silicic eruptions in different tectonic environments could help determine whether the reconstruction approach is appropriate for this dataset.

Considering our datasets in still more detail enables us to further parse the number of distinct magmas represented by the Highland Range rhyolites. The two Tuffs are similar in H_2_O and CO_2_, but they are distinguishable from each other outside of error (Fig. 6A). This suggests that the Upper Tuff A and Upper Tuff B eruptions represent distinct magmas. The high H_2_O and low CO_2_ in the Upper Tuff B relative to the Upper Tuff A is consistent with isobaric crystallization of the earlier-erupted Upper Tuff A magma driving compositions to higher H_2_O along the isobar (requiring ~10% crystallization). However, as discussed earlier, the trace element data indicate additional complexity. In the case of the Block-and-Ash and Obsidian samples, we see that the Block-and-Ash plateau inclusions extend to heavier δD_VSMOW_ values than the Obsidian inclusions (Fig. 7). However, the lack of large Obsidian plateau inclusions precludes us from further distinguishing the Block-and-Ash and Obsidian magmas. We therefore suggest the four rhyolitic eruptions tapped at least three, and potentially four, distinct preeruptive magmatic storage regions.

Our melt inclusion volatile data support a model in which the Highland Range rhyolite eruptions tapped a complex magmatic system with multiple discrete lenses of silicic, crystal-poor magma. If plateau H_2_O values are representative of the melt at the time of entrapment, then these rhyolites resided at consistently shallow depths before eruption, and they may have coexisted and been laterally juxtaposed or emplaced at different points in time to produce the distinct magmatic signatures we observe. If instead initial Block-and-Ash and Obsidian melt inclusion H_2_O concentrations were higher before slow cooling, then these magmas may have been stored at slightly greater depths than the Tuff magmas, producing a more vertically complex system of magma reservoirs.

### Eruptive processes

The difference in the eruptive style of the rhyolite units could reflect differences in the extent of degassing between the eruptions. However, there is no clear relationship between H_2_O degassing paths and eruptive style in our melt inclusion data (Fig. 9). In addition, degassing should deplete the melt in Cu, so the Cu enrichment we observe in the Block-and-Ash and Obsidian inclusions relative to the Tuffs is also inconsistent with expectations of progressive degassing (Fig. 5B). Instead, the differences in H_2_O, and potentially in δD_VSMOW_, between the various eruptions appear to be due to inherent differences in the magmas residing at depth, and the Block-and-Ash and Obsidian magmas are not the degassed products of either Tuff magma. Thus, the transitions from effusive eruptions to explosive eruptions in the Highland Range do not seem to have resulted from variable degrees of preeruptive magmatic degassing in a single eruptible magma body, as has been suggested for some other rhyolitic systems [e.g., Chaiten Volcano (*68*)].

If the plateau inclusions do preserve primary H_2_O contents of the Block-and-Ash and Obsidian magmas, the differences in the dissolved H_2_O contents of the various magmas may still have influenced their eruptive style by affecting bubble nucleation. Dissolved H_2_O has a pronounced effect on reducing melt viscosity [the effect of dissolved CO_2_ is comparatively minor; (*69*)], and the differences in melt inclusion H_2_O between our samples would qualitatively suggest that the Obsidian and Block-and-Ash melts were more viscous than those that formed the Tuffs. Viscosities determined using the model of Giordano *et al.* (*69*) differ by an order of magnitude: At 750°C, the viscosity of a 77.7 wt % SiO_2_ melt would be ~9.5 × 10^6^ Pa*s if it had 2.9 wt % dissolved H_2_O (highest plateau H_2_O) and ~1.3 × 10^6^ Pa*s with 4.2 wt % H_2_O (highest Tuff H_2_O). In this case, the high viscosity of the Obsidian and Block-and-Ash melts may have impeded eruption explosivity by inhibiting bubble nucleation (*70*) and/or enhancing the overpressure and energy required to initiate explosive eruptive behavior (*71*). Thermodynamic models also indicate that magmas with lower mole fractions of H_2_O may be more likely to erupt effusively than magmas with higher mole fractions of H_2_O, which are more likely to erupt due to internal overpressure from volatile exsolution (*72*). The comparatively high CO_2_ of the Block-and-Ash and Obsidian inclusions relative to the Tuffs would be consistent with this.

Our diffusive exchange models also provide insight into the ascent process for the various magmas. Our results suggest that magma decompression rates likely varied as the rhyolite eruptions progressed and transitioned in intensity from the Obsidian to the Tuffs to the Block-and-Ash flow (the Supplementary Materials). Some studies have suggested that differences in magma ascent rates can lead to differences in eruption explosivity even if initial volatile contents are similar (*73*). Within the framework of the Highland Range eruptive stratigraphy, it is notable that the Upper Tuff A inclusions do not show evidence of diffusive exchange, while the Upper Tuff B displays a subtle diffusive H_2_O loss signature, which we interpret as preeruptive ascent (increasing δD_VSMOW_ with decreasing H_2_O; Fig. 8C). This suggests that the Upper Tuff A magma ascended rapidly enough to prevent measurable diffusive equilibration, and the Upper Tuff B magma ascended more slowly. Thus, the decompression rates of the explosive eruptions apparently decreased as the eruption progressed from Upper Tuff A to Upper Tuff B. Future studies that incorporate hydrogen isotopes into estimates of magma ascent timescales will improve our understanding of magma decompression rates.

### Storage of multiple silicic magmas in the Searchlight Magmatic System

Decades of work support the idea that the Searchlight pluton is the intrusive counterpart to the Highland Range volcanic sequence (*31*–*33*, *54*) and was emplaced incrementally over time in relatively small volumes (*37*). The results of our study offer complementary insights from the volcanic perspective into the active evolution and variability of this system. Specifically, our datasets record the existence of several discrete batches of eruptible rhyolitic magma with limited interactions at depth. These magma batches may have intruded one by one into a common rhyolite storage region that drained and refilled over time, or they may have coexisted as juxtaposed magma bodies in a complex magmatic plumbing system. Regardless, both the eruptible and noneruptible portions of this magmatic system appear to have been emplaced incrementally in relatively small volumes. Our study highlights the power in combining information from melt inclusion volatiles, hydrogen isotopes, and trace elements, even from melt inclusions that have experienced postentrapment modifications, to gain perspectives on the preeruptive conditions in silicic magmatic systems.

Several juxtaposed magma bodies have been identified from interrogating the eruptive deposits of other silicic magmatic systems, including rhyolitic eruptions in the Taupō Volcanic Zone (*74*, *75*) and the Huckleberry Ridge Tuff eruption from Yellowstone Caldera (*76*). The Highland Range eruptions were considerably smaller volume eruptions than these examples, which involved 100s to 1000s of cubic kilometers of magma, but the geometric complexities suggested for large-volume magmatic systems may have also been present within this relatively small system (*77*). Future high-precision geochronology work on the rhyolite eruptions in this work could help determine whether these discrete rhyolitic bodies coexisted in the crust or whether a longer-lived dynamic storage region is more likely.

## MATERIALS AND METHODS

### Experimental design

The goal of this study is to characterize the trace element, volatile, and hydrogen isotope signatures of high-silica rhyolite eruptions, which exhibited distinct eruptive styles. We experimentally homogenized samples lacking glassy, homogeneous melt inclusions (Block-and-Ash sample 049a and Obsidian sample 047) in the laboratory to allow for analysis of glassy materials. Homogenization involves heating to melt daughter crystals and resorb vapor bubbles in the melt inclusion, then quenching to preserve the inclusion as glass. We performed quantitative analyses of volatiles, hydrogen isotopes, and trace element analyses in addition to geometric measurements on all samples and compared results to identify evidence for potential relationships between samples and evaluate the likelihood of several magmatic processes.

### Geologic setting and samples

The Miocene-age Searchlight Magmatic System is composed of the Searchlight pluton and the Highland Range volcanic sequence. The Searchlight Magmatic System is exposed in a ~15-km crustal section that was tilted up to ~90° along the east-dipping Dupont Mountain detachment fault in the Colorado River Extensional Corridor (Fig. 1). Field relationships, geochemistry, and geochronology [(*32*) and references therein] strongly suggest that some of the magma(s) that produced the Searchlight pluton and the Highland Range volcanic sequence were cogenetic. This study focuses on four high-silica rhyolite units of the upper Highland Range.

All four samples are saturated in quartz, sanidine, and plagioclase. The high-silica rhyolites are crystal poor, with up to 10 vol % crystals in Upper Tuff A and Upper Tuff B and 3 vol % crystals in the Obsidian and Block-and-Ash (file S3) (*32*). The presence of Obsidian lithics in the Upper Tuff A and B deposits, in addition to field relations, indicate that the eruption of the Obsidian flow occurred before the eruption of the pyroclastic flows that formed the Upper Tuffs (*78*). Stratigraphy suggests that the eruption of Upper Tuff A occurred before the eruption of Upper Tuff B, all of which is followed by the eruption of the Block-and-Ash flow (Fig. 1). Both the Upper Tuff A and Upper Tuff B units of this study are from the “Upper HSR Tuff” unit of the rhyolite section (*31*, *32*). The age of the entire silicic section of the Highland Range volcanic sequence is constrained by ~16.0-Ma underlying lavas and ~16.3-Ma capping lavas [Ar-Ar ages; (*34*)]. The rhyolites have been correlated with the leucogranite pod in the middle unit of the Searchlight pluton, which crystallized within ~90 ka (*36*, *37*).

### Sample preparation and melt inclusion homogenization

The matrix of Upper Tuff A and Upper Tuff B samples contains abundant lithics and potentially xenocrystic crystals. To avoid contamination by these materials, we scraped individual pumice clasts ~1 to 3 cm in diameter out of the tuff matrix using a dental pick (Fig. 2). We picked quartz grains (~250-μm to 2-mm diameter) containing glassy melt inclusions from these scraped pumice separates.

We gently crushed bulk samples of the Obsidian and Block-and-Ash with a wooden baseball bat to minimize grain fracturing before sieving and rinsing in water. We then picked quartz grains ≥250 μm in diameter containing devitrified melt inclusions from the sieved separates and heated them to homogenize the inclusions. We conducted the homogenization experiments in the Magma Dynamics Laboratory at the United States Geological Survey in Menlo Park, California, following techniques outlined by (*28*) after (*79*). We place the grains in crimped gold capsules and heated them to a temperature of 1004 (±2)°C over ~30 min in 1510 to 1530 bars of Ar pressure (i.e., a dry environment) in a Mo-Hf-Zr alloy assembly. We held samples at this condition for 30 min before quenching them to glass using pressurized air. Melt inclusions from the Tuffs did not require homogenization for quantitative analyses.

We selected quartz grains with glassy melt inclusions from all samples. We mounted the grains in epoxy and ground into them to enable viewing and imaging of melt inclusions on a petrographic microscope. Before exposing the melt inclusions for compositional analysis, we imaged each melt inclusion using a petrographic microscope. We used these images to estimate melt inclusion sizes and their distance to the edge of the host crystals. The scale of each image is calibrated for the magnification of the image. We used the maximum possible magnification for imaging each melt inclusion that kept the inclusion in optical focus (between ×50 and ×200 magnification). We then further ground into and polished the crystals to expose a portion of the melt inclusion glass for compositional analysis.

### Melt inclusion volume and distance to crystal edge estimates

We estimated melt inclusion volumes from our petrographic microscope images by fitting an ellipse to the inclusion in ImageJ and measuring the two axes of the ellipse for the length (*x*) and width (*y*) of the inclusion. We assume that the melt inclusions have an ellipsoidal geometry and use the average value of the *x* and *y* measurements for a given inclusion for the melt inclusion thickness *z* in the volume calculations. This approach is justified on the basis of comparison with direct spindle stage measurements of these melt inclusions (fig. S6). An assumption of ellipsoidal geometry can overestimate melt inclusion volumes relative to true three-dimensional measurements made with micro-CT (computed tomography), especially for large melt inclusions with volumes > 0.3 × 10^6^ μm^3^ (*80*). All melt inclusions considered in this study, with the exception of two inclusions from Upper Tuff A, fall below this size threshold; thus, we do not expect the assumption of ellipsoidal geometry to affect our overall conclusions. We measured the distance from the center of each melt inclusion to the closest crystal face in ImageJ. In cases where fragmentation made crystal faces unclear, we measured the distance to the closest fragmented edge. These distance measurements are therefore likely minima in many cases.

### Volatiles and hydrogen isotopes: Secondary ion mass spectrometry – IMS 1280

We measured volatiles (H_2_O, CO_2_, F, Cl, and S) and hydrogen isotopes by secondary ion mass spectrometry (SIMS) on the CAMECA IMS 1280 at the Northeast National Ion Microprobe Facility (NENIMF) at Woods Hole Oceanographic Institution across two analytical sessions. During the first session (Summer 2022), we analyzed the homogenized melt inclusions from the Block-and-Ash and Obsidian, and during the second session (Autumn 2023), we analyzed the naturally glassy melt inclusions from Upper Tuff A and Upper Tuff B. We mounted and gold coated the quartz grains in indium before SIMS analysis. We kept the 10-kV ^133^Cs^+^ primary ion beam small to avoid contaminating analyses with the host quartz; we achieved a spot size of ~7-μm diameter using a low primary beam current (~150 pA) in the initial Summer 2022 analytical session. We used a field aperture of 600 by 600 μm to block ions from outside the innermost 7.5 by 7.5 μm from the analysis crater. The second analytical session did not require the same degree of spatial resolution due to the size of the melt inclusions of interest, and thus, we used an ~15-μm-diameter spot to improve counting statistics in Autumn 2023 with a higher primary beam current (~300 pA). For both sessions, we applied a presputter time of 300 s at 500 pA with a ~10-μm raster before switching to either 150- or 300-pA beam for volatile analyses and applied no raster for analyses. This presputter preceded collection of ^12^C, ^16^OH, ^19^F, ^30^Si, ^32^S, and ^35^Cl, which we measured five times each. For D/H measurements, we opened the field aperture to 2000 by 2000 μm and did not apply a larger beam presputter or a raster. We measured ^16^OD and ^16^OH each over 75 cycles for D/H ratios at a mass resolving power (MRP) of ~9500. H_2_O is relatively high in rhyolites; the contribution of H_2_O from background is between 1‰ and 1% of the signal from the rhyolites, and thus, the background has a negligible effect on calculated D/H. We use a suite of standards including basalts, dacites, and rhyolites of known composition for calibrating volatiles to account for instrumental fractionation at various SiO_2_ contents, and we use a rhyolite glass of known composition (MCR2) for a D/H calibration. We measured counts using an electron multiplier. In the first analytical session, we recovered concentrations on the unknowns of H_2_O, CO_2_, F, Cl, and S with average 2σ uncertainties of 7, 22, 4, 8, and 13%, respectively, and we obtained average 2σ uncertainties of 4.7% on D/H ratios. In the second session, we measured H_2_O, CO_2_, F, Cl, and S on the unknowns with average 2σ uncertainties of 2, 66, 12, 14, and 10%, respectively, and 2σ uncertainties of 1.6% on D/H ratios. Volatile measurements depend on the SiO_2_ content in each melt inclusion; we applied a SiO_2_ content of 77 wt % SiO_2_ for these calibrations based on average major element measurements of the melt inclusions, but this assumption does not affect our interpretations (fig. S7). Repeat analyses of standards 519-4 and MCR2 throughout each SIMS analytical session reveal that during the 2023 session, analytical precision was on the order of 2% for D/H; 4% for H_2_O, CO_2_, F, and S; and 7% for Cl. Analytical precision during the 2022 session was on the order of 3% for S, 4% for F and D/H, 5% for Cl, 11% for H_2_O, and 33% for CO_2_ (all 2σ). These uncertainty differences are likely due to differences in counting statistics based on analytical spot size. Detection limits are on the order of <1 ppm of CO_2_, S, and Cl; 7 ppm of F; and 0.05 wt % H_2_O based on measurements of host quartz and background standard Supracil.

In their study of hydrogen isotopes in quartz-hosted melt inclusions, Befus *et al.* (*23*) observed a negative correlation in melt inclusion H_2_O-δD_VSMOW_ measured by SIMS that they interpreted as an analytical artifact resulting from matrix effects on ionization efficiency at different glass H_2_O contents. We do not expect this analytical artifact to affect our melt inclusion data or interpretations because Befus *et al.* (*23*) measured D and H ions on an IMS-6F, whereas we measured OD and OH on an IMS-1280. Repeat analyses of synthesized MCR glasses by IMS-1280 at the NENIMF facility reveal no comparable negative correlations between H_2_O and δD_VSMOW_ in rhyolitic glasses.

### Glass major elements: Electron probe microanalysis

We measured major elements in melt inclusion glasses on a JEOL JXA-8230 “superprobe” electron microprobe at the Stanford Mineral and Microchemical Analysis facility. Probe current was 2 to 20 nA depending on the element of interest, and accelerating voltage was 15 keV with a 5-μm beam size. On- and off-peak count times, spectrometer configuration, and primary calibrants are listed in file S4. Counts were collected in 10-s intervals for Si, Na, and K to monitor for elemental migration. A time-dependent intensity correction was applied for Na. Unknowns were bracketed by repeat analyses of secondary standard RGM-1. 2σ analytical precision on each measured element in RGM-1 is also reported in file S4.

### Glass trace elements: Sensitive high-resolution ion microprobe with reverse geometry

We measured trace elements in glass on the Stanford–US Geological Survey (USGS) sensitive high-resolution ion microprobe with reverse geometry (SHRIMP-RG) after IMS 1280 and electron probe microanalysis (EPMA) analyses. We avoided previous analysis spots when selecting spots on the SHRIMP-RG. We accelerated an O^2−^ primary ion beam with a spot diameter of 14 μm and depth of ~1 μm at 10 kV toward the samples with an intensity of ~1.5 nA. We did not analyze melt inclusions with smaller diameters than the SHRIMP-RG spot size. Analyses include measurements of ^7^Li^+^, ^19^F^+^, ^30^Si^+^, ^32^S^+^, ^35^Cl^+^, ^29^Si^16^O^+^, ^49^Ti^+^, ^54^Fe^+^, ^55^Mn^+^, ^63^Cu^+^, ^66^Zn^+^, ^69^Ga^+^, ^85^Rb^+^, ^88^Sr^+^, ^89^Y^+^, ^90^Zr^+^, ^93^Nb^+^, ^138^Ba^+^, ^139^La^+^, ^140^Ce^+^, ^146^Nd^+^, ^147^Sm^+^, ^151^Eu^+^, ^158^Gd^16^O^+^, ^159^Tb^16^O^+^, ^162^Dy^16^O^+^, ^166^Er^16^O^+^, ^172^Yb^16^O^+^, ^208^Pb^+^, ^232^Th^16^O^+^, and ^238^U^16^O^+^. Count times ranged from 3 to 12 s depending on the isotope being measured. We achieved MRP of *M*/Δ*M* = ~10,000 based on the 10% peak height on ^85^Rb. We ratioed count rates for each isotope to ^29^Si^16^O to account for primary current drift. We compared these ratios to those measured on several natural and synthetic standard glasses to determine unknown concentrations. Following (*28*), we preferred natural glass standards (Macusani, RLS-37, RLS-76-75, RLS-132, RLS-140, and RLS-158) for building calibration curves for each element analyzed, but where insufficient data exist for the natural glasses, we included synthetic National Institute of Standards and Technology (NIST) glasses (NIST-613 and NIST-611) in calibration curves. Standard compositions used are as reported in (*81*). We measured the full suite of standards on each day of analysis and interspersed several repeat measurements of RLS-140 with the unknowns to monitor instrument stability over time.

### Diffusive exchange modeling

We use the model from (*13*) for melt inclusion diffusive exchange calculations, which assumes a spherical melt inclusion located in the center of spherical crystal host. The density of the host quartz and the silicic melt inclusion are assumed to be 2.53 and 2.33 g/cm^3^, respectively. We use the H-in-quartz diffusion coefficient of (*21*). Melt inclusion radius is taken directly from petrographic measurements. A 10-μm-radius melt inclusion is considered representative of the smallest melt inclusions from the Block-and-Ash and Obsidian. The radius of the crystal host is taken from our measurements of distance to the closest crystal face; however, this parameter is not easily described in our samples due to the fragmentary nature of the crystals. With the exception of one Upper Tuff B melt inclusion, all melt inclusions in this study are located >50 μm from the closest crystal edge, so we considered 50 μm as the minimum host crystal radius for our diffusive exchange models. The extent of diffusive exchange in melt inclusions that are located this close to a host crystal edge is likely overestimated in our calculations due to the assumption of a symmetrical host crystal (i.e., 50 μm is the diffusion distance in all dimensions). In reality, melt inclusions that are located so close to a crystal edge are only within 50 μm of the edge in one dimension, not all dimensions. In such cases, we also test 75- and 100-μm crystal sizes in diffusive exchange models to account for this uncertainty. Input parameters and initial conditions of each diffusive exchange model are described in the Supplementary Materials and listed in file S2.

### Statistical analysis

The IQR was calculated for the volatile concentrations of each sample to identify outliers. Compositions lying outside of 1.5× IQRs are considered outliers and identified as such. All reported uncertainties and SDs are 2σ (95% confidence interval).

## Supplementary Materials

### The PDF file includes:

Supplementary Text Figs. S1 to S7 Legends for data S1 to S5 References
