# Supplementary material for: Reliability of quartz-hosted melt inclusions and discrete rhyolite reservoirs revealed by H isotopes, trace elements, and volatiles
Source: Sci Adv. 2026 Mar 13;12(11):eadw1667. doi: 10.1126/sciadv.adw1667 (PMC12985731; doi:10.1126/sciadv.adw1667)
Supplement: Supplementary file 1 — Supplementary Text Figs. S1 to S7 Legends for data S1 to S5 References [file sciadv.adw1667_sm.pdf]

Science Advances
AAAS

Supplementary Materials for

Reliability of quartz-hosted melt inclusions and discrete rhyolite reservoirs revealed by H isotopes, trace elements, and volatiles

Sarah M. Hickernell *et al.*

Corresponding author: Sarah M. Hickernell, smhick@uw.edu

Sci. Adv. **12**, eadw1667 (2026)
DOI: 10.1126/sciadv.adw1667

The PDF file includes:

Supplementary Text
Figs. S1 to S7
Legends for data S1 to S5
References

Other Supplementary Material for this manuscript includes the following:

Data S1 to S5

Supplementary Text

Models: Approach and Results

Quartz resorption from homogenization procedure

Homogenization of Block-and-Ash and Obsidian melt inclusions may have induced dissolution of the host quartz, resulting in the observed SiO_2 enrichment at small melt inclusion sizes in these samples. To assess if this is the case, we used a mass balance calculation. Melt inclusion dilution by melting and assimilation of host quartz assumes a starting composition of 76 wt. % SiO_2, which is within error of the lowest SiO_2 melt inclusion from the Block-and-Ash (049a-g13a). Except for one Obsidian melt inclusion with >90 wt.% SiO_2, which we exclude, the maximum observed melt inclusion SiO_2 content is 83.6 wt.% in a small, homogenized inclusion (e.g., 047-g15). We model the relative mass of quartz assimilated into a melt inclusion assuming the largest potential compositional change, from 76 to 84 wt. % SiO_2. The maximum SiO_2 enrichment we see in the data is 8 wt. %, and we estimate that homogenization would need to melt a mass of quartz equivalent to 0.33 of the original inclusion to achieve this enrichment. This corresponds to a maximum dilution factor of 33% for all other elements in the homogenized melt inclusions and is most relevant for the smallest melt inclusions.

CO_2 enrichment from H_2O loss

H_2O loss from a melt inclusion leads to enrichment of other melt components, like CO_2. Our calculation of CO_2 enrichment due to melt inclusion H_2O loss utilizes the major element, H_2O, and CO_2 concentrations of Upper Tuff A melt inclusion 046-g15-a (3.8 wt.% H2O, 65 ppm CO_2). Our mass balance calculations indicate that the CO_2 content of an Upper Tuff A melt inclusion would increase by ~4% if the inclusion was completely dehydrated through diffusive loss. This is within error of our CO_2 measurements.

Fractional crystallization

Fractional crystallization models are performed using partition coefficients for Ba and Sr calculated for the Peach Spring Tuff (82) and assuming a Rayleigh fractionation model. The least-evolved melt inclusion compositions for the Block-and-Ash, the Block-and-Ash Plateau, Obsidian, and Upper Tuff A were selected based on their high Ba contents, a compatible element in feldspar-saturated high-silica rhyolites. For the Upper Tuff B, the inclusion with the second-highest Ba (3 ppm) was selected because the inclusion with the highest Ba (4 ppm) has lower Sr than most of the other Upper Tuff B melt inclusions. The two modelled crystal assemblages are based on our own petrographic observations of each sample's mineral assemblage, petrographic observations from (35), and the mineral assemblage calculated for silicic Searchlight magmas from (32) from Rhyolite-MELTS crystallization simulations. The two mineral mode endmembers tested include: A) 0.50 quartz, 0.42 sanidine, 0.05 plagioclase, 0.03 biotite; and B) 0.39 sanidine, 0.26 plagioclase, 0.25 quartz, and 0.10 biotite. The range in reported crystallization resulting from our models (e.g. 50-70% crystallization) reflects the variability from applying Assemblages A and B. These two assemblages are considered endmember

scenarios and generally capture most observed variability in melt inclusion trace element compositions.

Fractional crystallization models in Ba-Sr space from the highest-Ba Block-and-Ash melt inclusion (049a-g22a) demonstrate that approximately 50-70% crystallization is required to reproduce the full range of Ba and Sr in the Block-and-Ash melt inclusions (Fig. 3C). The Block-and-Ash plateau inclusions alone represent ~10% crystallization from the highest Ba plateau inclusion (049a-g13a). Models from high-Ba Obsidian (047-g15), Upper Tuff B (026-g13), and Upper Tuff A (046-g23) inclusions suggest the melt inclusion compositions represent 40-60%, 10-20%, and 20-30% fractional crystallization, respectively (Fig. 3). Our models suggest ~50-60% fractional crystallization would be required to produce the lowest Ba and Sr compositions amongst our samples (Upper Tuff B) from melt inclusions with the highest measured Ba and Sr contents (Block-and-Ash inclusion 049a-g22a).

Diffusive Exchange: Laboratory Homogenization-Induced

We model diffusive H_2O exchange utilizing the conditions from laboratory homogenization to test whether the observed inter- and intrasample variation we observe is due to the laboratory homogenization process. Diffusive H_2O loss during laboratory homogenization is calculated utilizing 0 wt% external H_2O and external δD_{VSMOW} = 0 ‰ for a 45-minute duration, accounting for experimental temperature ramp-up and cool-down time after Rout et al. (83), at 1004 °C (see File S5). The Tuff melt inclusion compositions and geometries selected to model endmember scenarios for laboratory-induced diffusive H_2O loss include that of the isotopically heaviest and driest analyzed tuff melt inclusion (046-g24a), the smallest tuff inclusion (026-g1), an outlier inclusion (026-g23), and a low H_2O but isotopically light melt inclusion from Upper Tuff A (046-g26a). The initial condition selected to reproduce the range in plateau H_2O-δD assumes 10 μm melt inclusion radius, 50-100 μm host crystal radius, and initial H_2O and δD of 2.9 wt.% and 0‰, respectively.

Model results utilizing homogenization conditions (1004 °C, 150 MPa, 45 minutes) suggest that the Tuff melt inclusions would still contain more H_2O than the Block-and-Ash and Obsidian plateau inclusions after subjection to laboratory homogenization (Fig. 6A). In fact, no models of lab-induced diffusive loss using realistic inputs of melt inclusion size and position reproduce the plateau data from the main Tuff populations. Only very small (~10 μm radius) homogenized melt inclusions with initial Tuff-like compositions begin to approach Block-and-Ash plateau compositions in H_2O-δD_{VSMOW} space (File S2). However, this is not compatible with the large sizes of the plateau inclusions and cannot explain the inter-sample variability. After homogenization, a small melt inclusion within ~100 μm of the crystal edge with initial plateau-like H_2O-δD would have a similar signature to the isotopically heaviest Block-and-Ash melt inclusions.

Our models using the H_2O and δD_{VSMOW} of an Upper Tuff B outlier inclusion (026-g23), which is notably H_2O-poor, CO_2-rich, and one of the isotopically heaviest Tuff inclusions measured, suggest that this inclusion would have ~2.7 wt% H_2O and δD_{VSMOW} = -6 to -9‰ after homogenization; the range reflects our assumptions about the position of the inclusion (Fig. 6A; see Methods). These values are comparable to the isotopically lightest measurements from the Block-and-Ash and Obsidian plateau, which might suggest that the homogenized inclusions had similar initial compositions to the Upper Tuff B

outlier 026-g23. However, this model cannot account for the isotopically heavier plateau compositions, and there are no other compelling compositional relationships that are consistent with this possibility. Obsidian lithics are frequently observed in the bulk tuff matrix material in the Highland Range, and we may have introduced an errant quartz grain from a lithic into the separated material from the Upper Tuff B during sample preparation. Alternatively, inclusion 026-g23 may reside in a quartz grain that originally grew in a different magma and was entrained in the Upper Tuff B magma prior to its eruption, which we explore in greater detail in the next section.

The isotopically heaviest Block-and-Ash inclusions not on the high-H_2O plateau are compatible with a laboratory homogenization-induced diffusive H_2O loss model (Fig. 6A), but the range of δD_{VSMOW} in the Block-and-Ash plateau inclusions cannot be reproduced through modelling any diffusive H_2O exchange process. Diffusive exchange that produces the ~60‰ increase in δD_{VSMOW} observed across the plateau would have a large (~0.8 wt. %) associated reduction in H_2O, which we do not observe. Instead, the Block-and-Ash plateau melt inclusions form a near vertical array in H_2O-δD_{VSMOW}.

Diffusive exchange of Li between a melt inclusion and its external environment might occur during heating experiments given its rapid diffusivity in quartz (21). Experimental homogenization occurred in a Li-free environment, suggesting diffusive loss of Li might occur. For Li diffusion calculations, an Arrhenius relationship was fit to the Li-in-quartz diffusivity data of Jollands et al. (50). This corresponds to the equation $\ln(D) = -15.764 - 11045/T$. We find that at 1000 °C, Li diffusivity in quartz is on the order of 2.3×10^{-12} m/s^2. We perform this calculation assuming a melt inclusion radius of 10 μm and host crystal radius of 50 μm to approximate the greatest extent of potential diffusive Li equilibration during homogenization. Our models suggest that small melt inclusions (10 μm radius) 50 μm from the host crystal edge will experience the greatest extent of diffusive loss but still retain ~87% of their original Li after homogenization. Even melt inclusions with more dramatic, unrealistic geometries (e.g., 15-μm radius inclusion in a 20-μm radius host crystal) would retain ~75% of their original Li through our laboratory homogenization (File S2).

Diffusive Exchange: Entrainment of melt inclusion-bearing xenocrysts

If a melt inclusion-bearing quartz crystal containing melt from the Obsidian or Block-and-Ash magma(s) resided in the Upper Tuff B melt for some period prior to eruption, as may be the case for outlier Upper Tuff inclusion 026-g23, diffusive exchange could occur between the xenocrystic melt inclusions and the hydrogen isotopic reservoir of the Upper Tuff B melt. Diffusive exchange during entrainment of a plateau melt inclusion in the Upper Tuff B magma assumes a 750 °C, 4.2 wt. % H_2O, and δD_{VSMOW} = -65 ‰ external environment, based on the highest melt inclusion H_2O measured in this sample. A typical plateau melt inclusion initial composition is assumed to be 2.86 wt. % H_2O and δD_{VSMOW} = 0 ‰. Our models suggest it would take ~14 hours at 750 °C to produce a H_2O-δD_{VSMOW} signature similar to that observed in melt inclusion 026-g23 (Fig. 6B) from initial plateau values. Varying the host crystal size from 50 to 100 μm extends the range of this timescale from 13 to 15 hours. Consequently, entraining a melt inclusion-bearing quartz from the Obsidian or Block-and-Ash magma(s) into the Upper Tuff B magma ~14 hours before the Upper Tuff B eruption appears to be a reasonable explanation for the anomalous composition of 026-g23. While this quartz grain may have been

transferred from the Obsidian or Block-and-Ash magma(s) into the Upper Tuff B magma when both magmas were mobile at depth (within one day of eruption), other evidence for interaction and mixing between the various rhyolitic magmas at depth is limited.

Although it is an outlier in the Upper Tuff B dataset, melt inclusion 026-g23 could also provide important insights into the reliability of quartz-hosted melt inclusions and the architecture of the rhyolite reservoirs. First, this Upper Tuff B inclusion – which was naturally glassy and did not require homogenization – closely resembles the compositions of the homogenized Block-and-Ash and Obsidian plateau melt inclusions. This supports our previous contention that the volatile contents of large, devitrified quartz-hosted melt inclusions can withstand the laboratory homogenization process. Second, the hydrogen isotopic signature of this outlier is consistent with the entrainment of a melt inclusion-bearing quartz crystal from the Block-and-Ash or Obsidian in the Upper Tuff B magma for approximately 14 hours (Fig. 8B). This suggests these discrete rhyolitic magmas coexisted in the shallow crust for at least some period of time prior to their eruptions. If this quartz grain came from the Block-and-Ash or Obsidian magmas but did not have time to diffusively equilibrate with the host Upper Tuff B magma upon entrainment, its H_2O and δD_{VSMOW} could record that of the initial Block-and-Ash or Obsidian magma unaffected by homogenization and/or slow cooling.

Diffusive Exchange: Entrainment of melt inclusion-bearing xenocrysts

If the Block-and-Ash and Obsidian cooled slowly upon emplacement at the surface after eruption, their quartz-hosted melt inclusions may have experienced diffusive H_2O loss to the hot degassed surrounding matrix material. Our models suggest that the initial H2O content of the plateau melt inclusions would have been between ~3.8 and 5.5 wt. % assuming the initial δD was similar to the isotopically lightest measured inclusions (-65‰).

Diffusive Exchange: Slow post-eruptive cooling

The devitrification of Block-and-Ash and Obsidian melt inclusions larger than ~20 μm in diameter suggests that these eruptive units cooled slowly after eruption relative to the Tuffs. We model whether the Block-and-Ash and Obsidian plateau melt inclusion H_2O-δD compositions can be reproduced by diffusive H_2O loss during a simplified history of slow post-eruptive cooling.

We assume that the external melt upon post-eruptive emplacement contained 0.5 wt.% H_2O, which is a reasonable value for degassed matrix glass and comparable to the lowest measured melt inclusion H_2O contents. The δD_{VSMOW} of the external degassed melt would be between -100 and -125 ‰ based on our closed- and open-system degassing models, respectively, for the Tuff melt inclusions. By assuming a temperature condition equivalent to the glass transition temperature, we can calculate the maximum timescale over which the melt inclusions may have experienced post-eruptive H_2O loss. The glass transition temperature is traditionally assumed to occur at a viscosity of ~10^{12} Pa*s (69). Assuming a melt major element composition comparable to the matrix glass of the Block-and-Ash and 0.5 wt.% H_2O, the glass transition temperature should be ~640 °C (Giordano et al. (69) viscosity model). We thus assume an external condition of 0.5 wt. % H_2O and δD_{VSMOW} = -100 and -125 ‰, at 640 °C. In nature, a range of intermediate temperatures between pre-eruptive storage conditions (~700-750°C) and the glass transition temperature

will occur after emplacement, the consideration of which would result in faster diffusivities and shorter cooling timescales. The emplacement cooling timescales of these eruptions are not the focus of this effort, so we do not attempt to more precisely describe the cooling history – we are primarily concerned with the diffusive loss path of melt inclusions in H_2O-δD space in this context. We assume an initial δD_{VSMOW} in the block and ash and obsidian melt inclusions of -65‰, which is the isotopically lightest measured in our dataset. This is likely an oversimplification of primary magmatic δD (see Discussion). We use these constraints to model the initial H_2O of the block and ash and obsidian melt inclusions by testing a range of timescales over which the block and ash and obsidian cooled at the surface after eruption to produce the observed plateau compositions.

We select the largest Block-and-Ash plateau melt inclusion, 049a-g13, which also contains the highest measured H_2O (2.9 wt. %) in this sample, for modelling. A melt inclusion with its geometry (40 µm inclusion radius, 196 µm crystal radius) with an initial δD_{VSMOW} of -65‰ would have contained ~4.2 wt.% H_2O prior to ~35 days of cooling at 640 °C (see Fig. A below). This result could suggest that the initial H_2O-δD of this Block-and-Ash melt inclusion may have resembled that of the Tuffs prior to emplacement, however the CO_2 and F enrichment of the block-and-ash melt inclusions relative to those from the tuffs still cannot be explained by this diffusive loss process. If initial H_2O was lower (~3.8 wt. % H_2O), after slow cooling, its H_2O-δD would resemble the plateau melt inclusions with slightly lower H_2O values. We also model diffusive loss from the isotopically heaviest Block-and-Ash plateau melt inclusion (049a-g3). Assuming its radius of 30 µm and host crystal radius of 97 µm, the measured composition of this melt inclusion would have required ~5.5 wt. % H_2O initially prior to ~30 days of cooling at 640°C if its starting δD = -65‰. If this H_2O-δD reconstruction approach is appropriate, this suggests that Block-and-Ash and Obsidian melt inclusions could have originally contained between ~3.8 and 5.5 wt.% H_2O. Their H_2O-CO_2 volatile saturation pressure estimates would be shifted deeper - on the order of ~200-250 MPa rather than the ~100-150 MPa estimated from measured H_2O-CO_2 contents.

Ascent and Decompression

During magma ascent, melt H_2O will decrease due to its pressure-dependent solubility, which could provide a compositional gradient for diffusive H_2O loss from melt inclusions entrapped prior to ascent. Rapid ascent would induce less diffusive H_2O loss relative to slow ascent.

Ascent rate calculations assume an average crustal density of 2700 kg/m^3 after Wallrich et al. (32). External H_2O is assumed to be either 1.05 wt% (lowest measured Block-and-Ash H_2O), 0.57 wt.% (lowest measured Obsidian H_2O), or 0.1 wt% (typical H_2O content for a degassed Obsidian matrix glass). External δD_{VSMOW} was taken from the same low H_2O Block-and-Ash and Obsidian melt inclusions. For the 0.1 wt.% external H_2O cases, we varied external δD_{VSMOW} (0‰, -100‰, -200), however the resulting H_2O and δD_{VSMOW} signatures, as well as calculated timescales, are insensitive to external δD_{VSMOW} over ascent timescales (i.e. hours; File S2). Eruptive ascent is rapid enough such that melt inclusion δD_{VSMOW} always becomes initially isotopically heavier during ascent, even when the external environment is isotopically lighter than the inclusion. Nearly all magmatic H_2O must be lost to degassing before the choice of external δD_{VSMOW} becomes important for our ascent calculations.

If the Upper Tuff B magma incorporated the xenocrystic quartz grain 026-g23 from the Obsidian flow while passing through the volcanic pile during ascent, the calculated 14-hour timescale from our entrainment diffusive exchange model could reflect the Upper Tuff B magma ascent time. This timescale estimate assumes the Upper Tuff B magma did not lose extensive H_2O on its path to the surface, which is likely an oversimplification. If the host Upper Tuff B magma underwent extensive ascent-driven degassing after initial entrainment of the xenocrystic quartz, the host magma would have rapidly lost H_2O and gradually approached isotopically lighter δD_{VSMOW}. This would effectively lessen the chemical potential gradient driving diffusion, and thus this estimated timescale from this outlier melt inclusion may slightly underestimate its true ascent timescale. Assuming the Upper Tuff B resided at 100 MPa prior to a 14-hour ascent corresponds to a decompression rate of 0.0019 MPa/s (ascent rate of 0.075 m/s, assuming an average crustal density of 2700 kg/m^3 after (32)). This decompression rate is comparable to those estimated for other rhyolitic volcanic systems but is at the slow end of other estimates for explosive eruptions (84).

The H_2O-δD_{VSMOW} trend we observe in the main population of Upper Tuff inclusions could be the result of compositional variability in the magma during melt inclusion entrapment, however it is also consistent with H_2O loss during ascent (heavier δD_{VSMOW} at lower measured H_2O; Fig. 6C). Magma ascent times estimated from the H_2O-δD_{VSMOW} trends of this population are 7-9 hours, depending on the external H_2O concentration (see Methods; File S2). This corresponds to an Upper Tuff decompression rate between 0.004 and 0.003 MPa/s (ascent rates between 0.15 and 0.11 m/s), which is nearly twice as fast as the ascent rate we estimate for the outlier melt inclusion 026-g23.

Degassing

We calculated H_2O-CO_2 isobars, degassing paths, and melt inclusion H_2O-CO_2 saturation pressures using the MagmaSat (85) calibration within VESIcal (86). We calculated saturation pressures assuming a magmatic temperature of 800 °C, the lowest temperature for which this model has been calibrated, utilizing the major element composition measured for each melt inclusion by EPMA-WDS. We calculated H_2O-CO_2 isobars using the same representative melt inclusion major element composition used to calculate volatile concentrations. We performed H_2O-δD_{VSMOW} degassing models in the program VolcDeGas (87) which has been calibrated for rhyolitic systems. We assumed a magmatic temperature of 700 °C; lower temperatures lead to greater fractionation, so these model results can be considered maximum degassing fractionation values.

Calculated H_2O-CO_2 saturation pressures are similar for all samples, so there is no evidence that melt inclusions were entrapped during degassing due to decompression. However, we model the effects of degassing for comparison. Deuterium preferentially enters the vapor phase during exsolution of a magmatic H_2O vapor phase, leading to isotopically lighter melt (more negative δD_{VSMOW} values) as degassing proceeds. In open system degassing, where the magmatic vapor phase leaves the system after exsolution, melt δD_{VSMOW} fractionates to a greater degree than in closed system degassing, where the exsolved vapor phase remains in contact with the melt and maintains equilibrium. The closed-system degassing case buffers the melt from reaching as negative δD_{VSMOW} values as in the open-system case. In either degassing scenario, the melt contains progressively lighter hydrogen isotopes as magmatic degassing proceeds.

While the Block-and-Ash and Obsidian melt inclusions contain lower H_2O than those from Upper Tuff A and Upper Tuff B, their δD_{VSMOW} and CO_2 compositions are inconsistent with entrapment of a degassing Upper Tuff A or Upper Tuff B magma (Fig. 7). The differences in H_2O and CO_2 between the Block-and-Ash flow and Tuff inclusions are not explained by either open- or closed-system H_2O-CO_2 degassing models. In particular, the vertical trend of the plateau inclusions in H_2O-CO_2 follows a degassing curve from the highest CO_2 Block-and-Ash plateau inclusion, but they are not consistent with degassing trends in H_2O-δD_{VSMOW}. In contrast, two Upper Tuff B low H_2O outliers fall on degassing paths from initial Tuff compositions in both H_2O-δD_{VSMOW} and H_2O-CO_2 space (Fig. 7). However, these inclusions are also depleted in Rb compared to the rest of the Upper Tuff B inclusions, which conflicts with entrapment during degassing, if the Upper Tuff B melt was also crystallizing (File S1). The outliers are also depleted in Sr relative to the rest of the Upper Tuff B melt inclusions and do not fall on the modelled crystallization trends (Fig. 3E). This suggests the low H_2O outliers in the Upper Tuff B are likely xenocrystic and we do not consider them further.

The compositions of most Obsidian inclusions can be explained by diffusive loss and/or decrepitation, but it is plausible that the lighter isotopic values – relative to Block-and-Ash inclusions – are the result of magmatic degassing. A melt with an initial plateau composition would approach the non-plateau H_2O-δD_{VSMOW} Obsidian inclusion compositions (Fig. 7B).

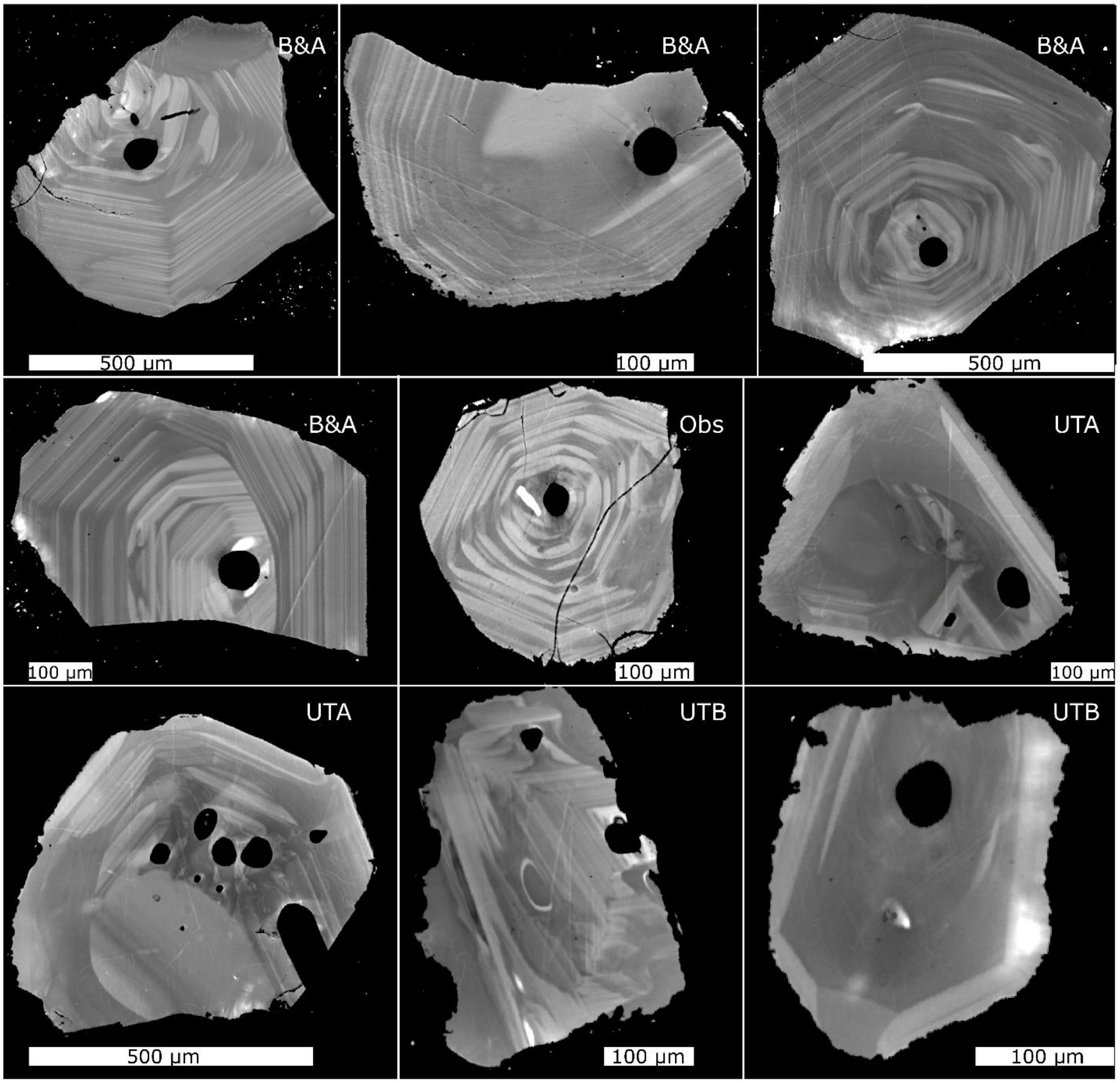

Fig. S1.

Representative Quartz Cathodoluminescence (CL) maps. Each grain is labelled by its unit of origin: B&A – Block-and-ash; Obs – Obsidian; UTA – Upper Tuff A; UTB – Upper Tuff B. Each grain image has its own scale bar in white. The melt inclusions do not emit a strong CL signal and appear as black ellipses. Note the complexity of zoning in quartz from all units. Images collected with a hyperspectral CL detector on the JEOL JXA-8230 "superprobe" electron microprobe at the Stanford Mineral and Microchemical Analysis facility.

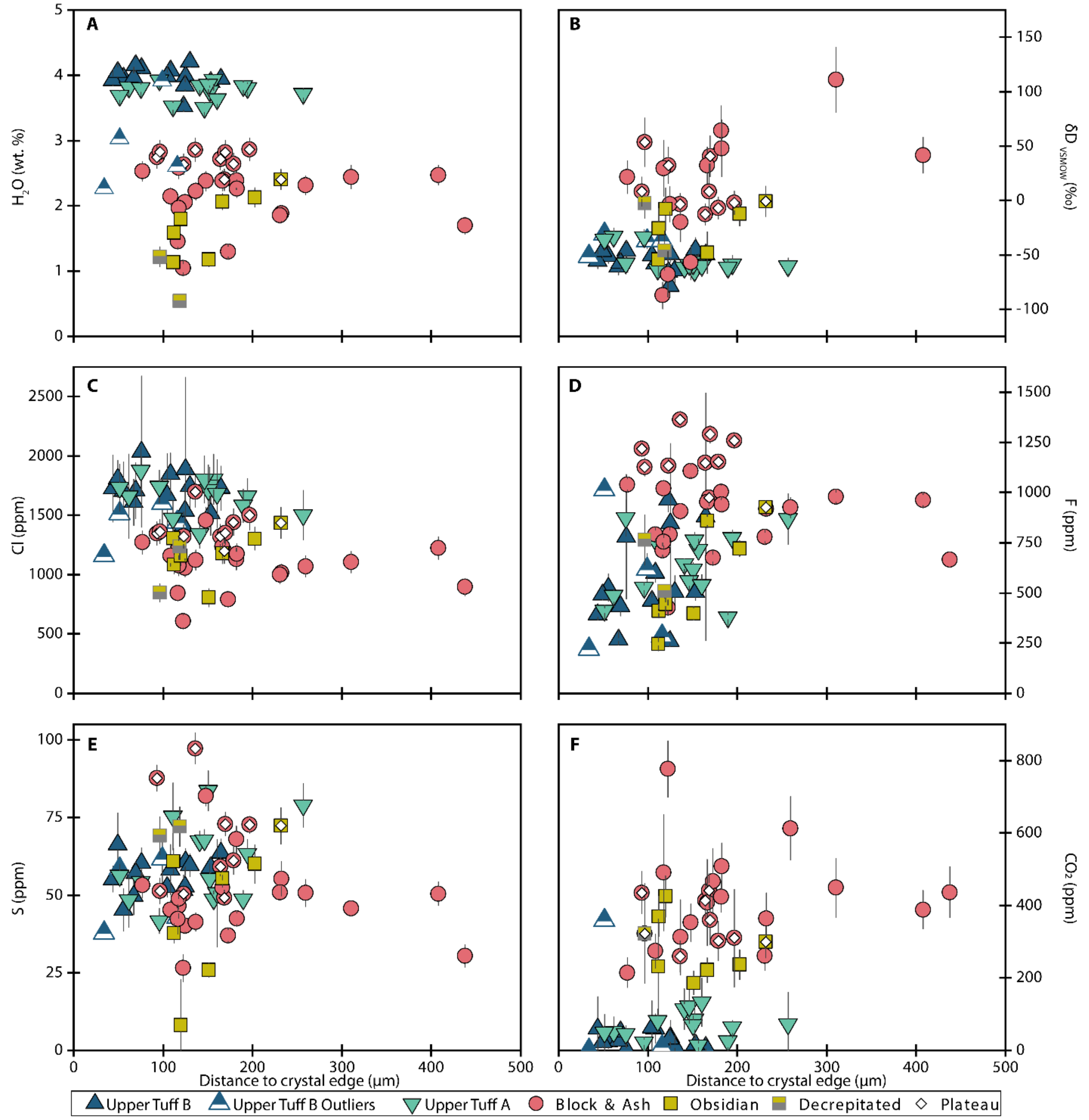

Fig. S2.

Melt inclusion distance from crystal edge vs. volatile concentrations. Error bars are 2σ or smaller than symbol size. Panels display relationships with (**A**) H_2O, (**B**) δD_{VSMOW}, (**C**) Cl, (**D**) F, (**E**) S, and (**F**) CO_2.

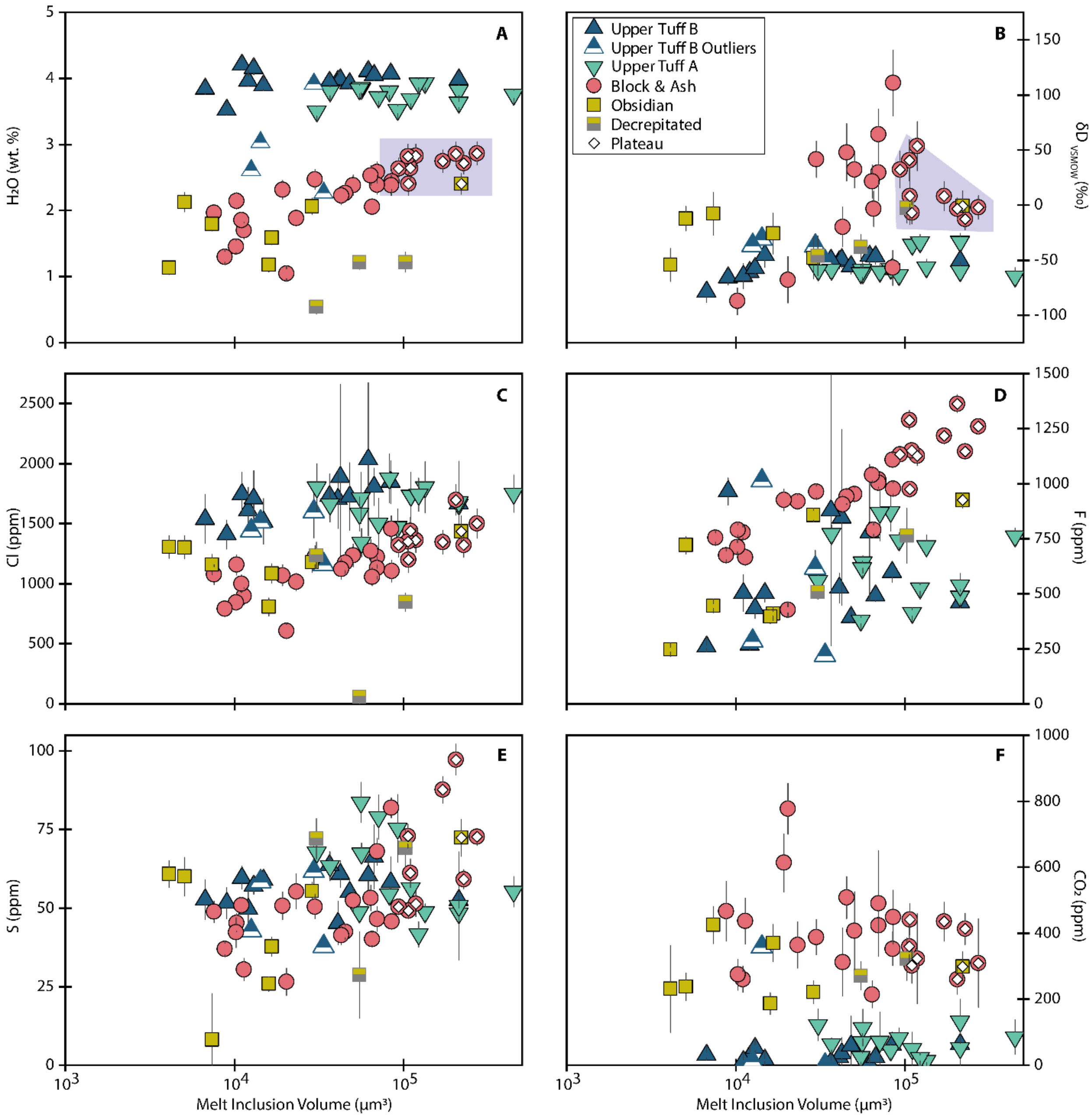

Fig. S3.

Melt inclusion volume vs. volatiles in log scale. This version of Fig. 4 provides more insights into the compositions of the smallest melt inclusions. Panels display relationships with (**A**) H_2O, (**B**) δD_{VSMOW}, (**C**) Cl, (**D**) F, (**E**) S, and (**F**) CO_2. All error bars are 2σ or smaller than symbol size.

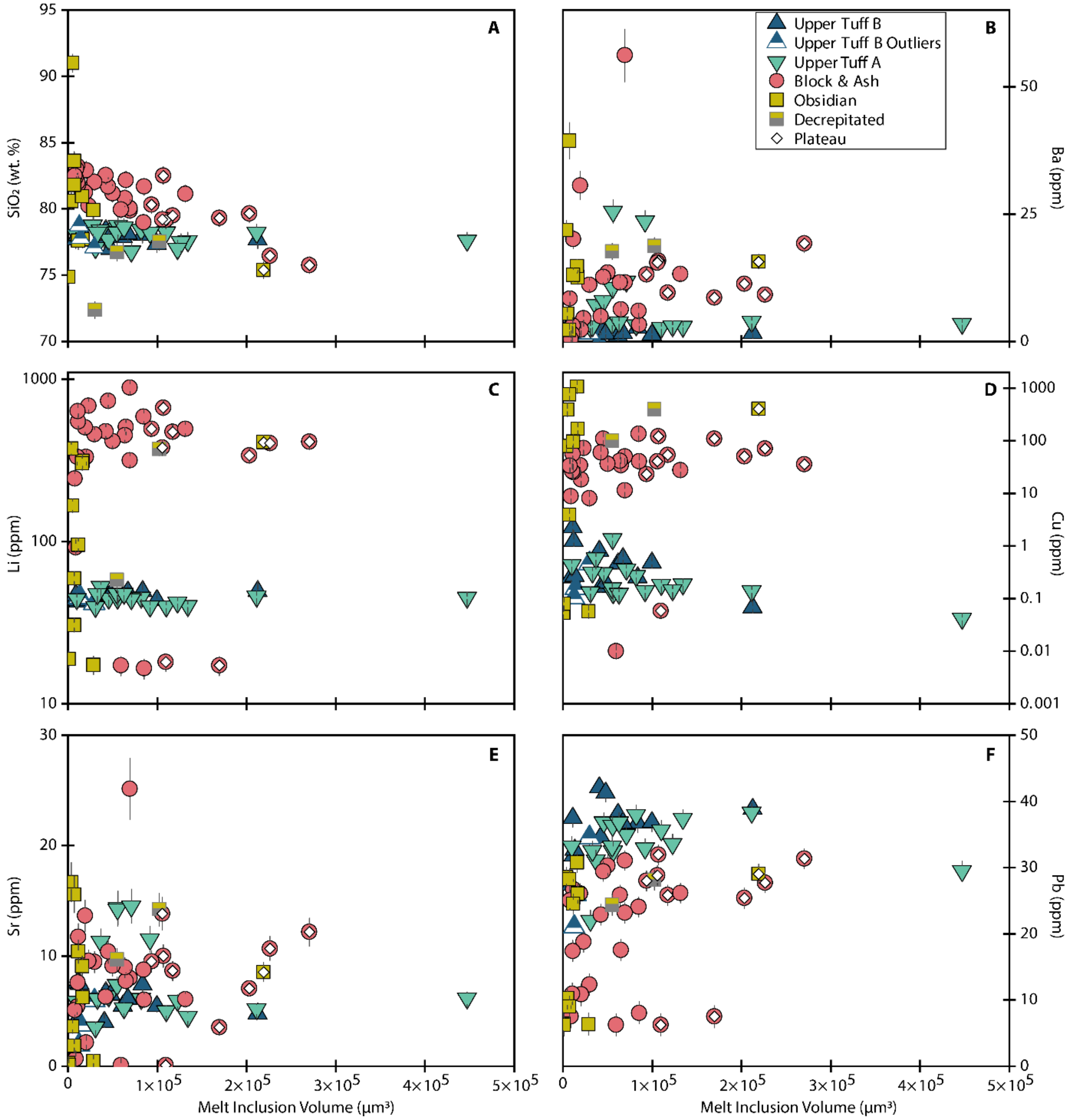

Fig. S4.

Melt inclusion volume vs. major and trace element concentrations. (**A**) SiO_2, (**B**) Ba, (**C**) Li, (**D**) Cu, (**E**) Sr, and (**F**) Pb. All error bars are 2σ or smaller than symbol size.

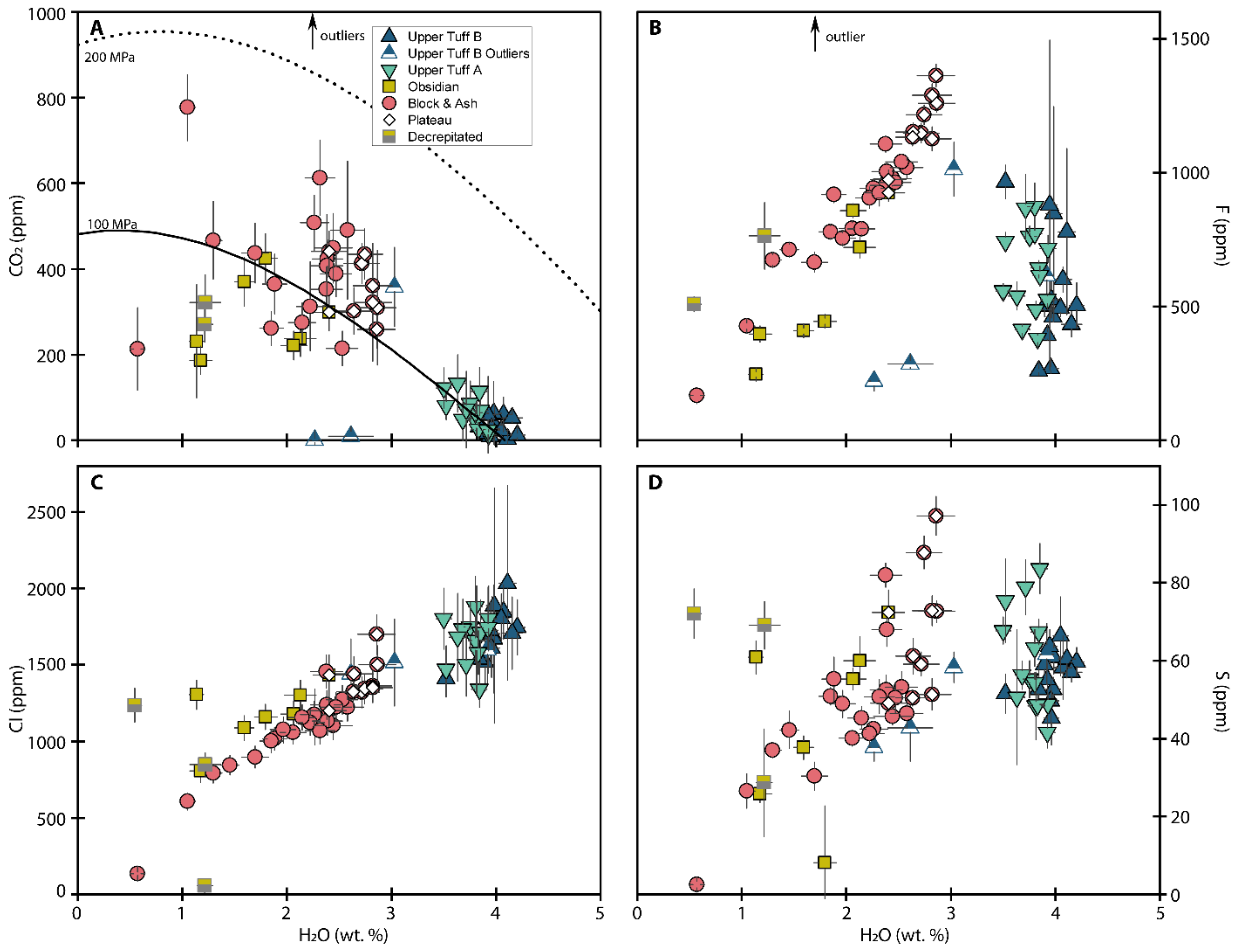

Fig. S5.

All melt inclusion volatile data. Plot includes small, homogenized melt inclusions that do not fall on the plateau in Fig. 4. Panels display H_2O vs. (**A**) CO_2, (**B**) F, (**C**) Cl, and (**D**) S. All error bars are 2σ or smaller than symbol size.

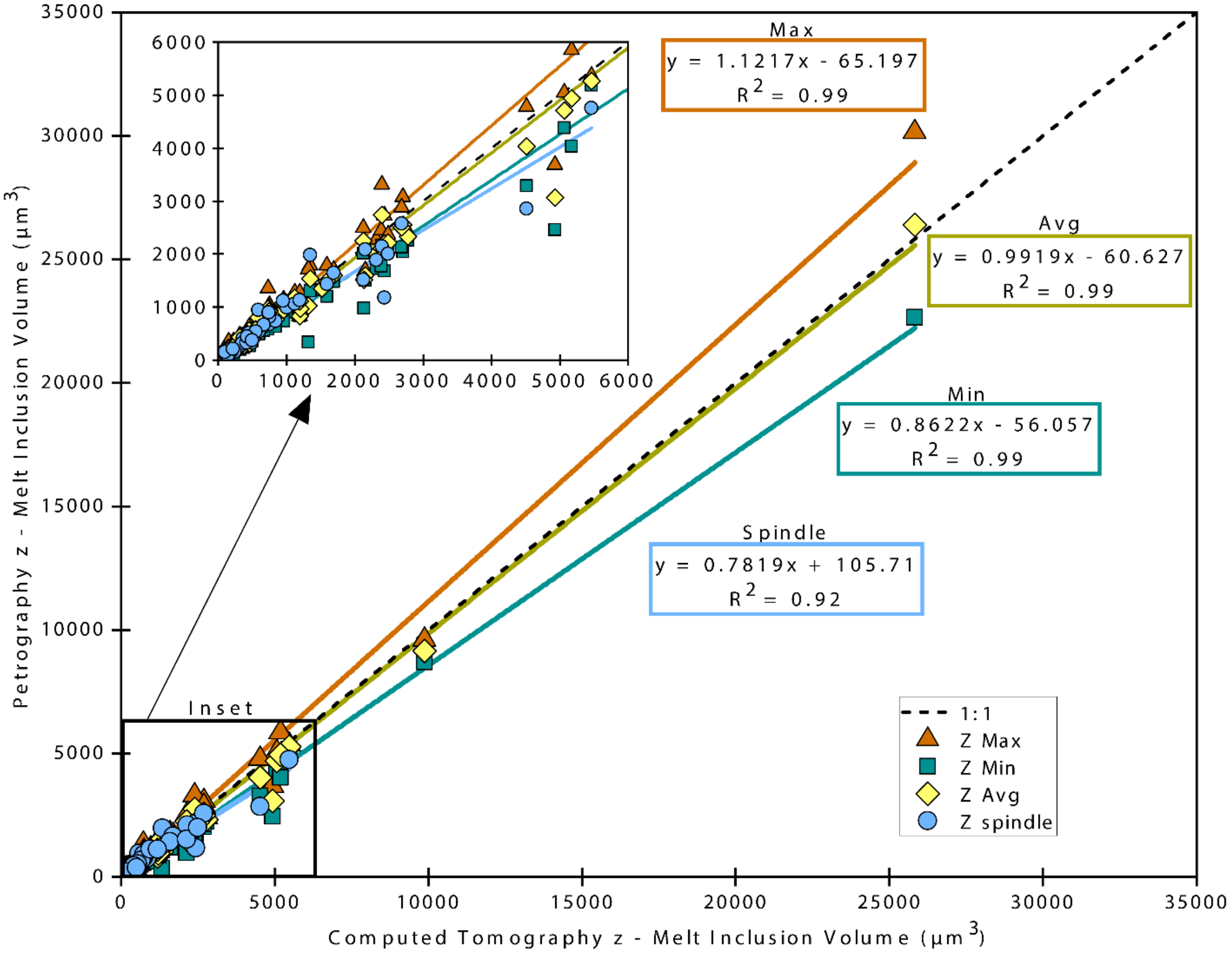

Fig. S6.

Comparison of melt inclusion volume estimates. Volume estimates derived from petrographic estimates of melt inclusion height, z, and measurements of z from computed tomography (CT). Imaging conditions are as reported in (88). All volumes are calculated assuming an ellipsoidal geometry. CT z measurements were made by counting the number of voxels between the top and bottom of each melt inclusion and using the voxel resolution to determine melt inclusion z. Spindle refers to spindle stage. These measurements were made by mounting grains on toothpicks parallel to the c-axis and placing them on their side (i.e., c-axis parallel to surface) in a well filled with a sugar solution with a refractive index of ~1.54 (to hide adhering glass). This enabled the inclusion to be viewed perpendicular to the c-axis of the crystal and measurement of the z-axis of the inclusion. Avg refers to using an average value of x and y for the z measurement. MinXY utilizes the minimum value of x and y for z, and MaxXY uses the maximum value of x and y. A linear equation is fit to each petrographic method. The inset panel displays results for the smallest measured melt inclusions on a smaller scale. Utilizing the average of melt inclusion x and y values for z provides melt inclusion volume estimates most consistent with CT volume estimates (slope = 0.99).

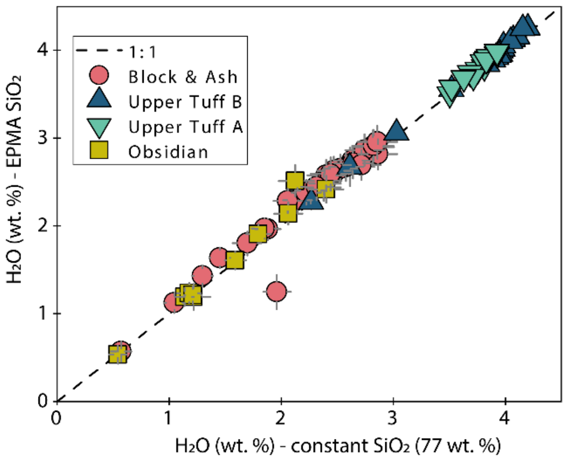

Fig. S7.

Effect of glass major element composition on calculated H_2O concentrations. The x-axis provides results using the same SiO_2 value (77.7 wt. %) for all melt inclusions. The y-axis provides results using the SiO_2 value measured for each individual inclusion. The black line denotes the 1:1 line between estimates. All error bars are 2σ or smaller than symbol size.

Data S1. (separate file)
Melt inclusion data, including sizes, major element, trace element, and volatile compositions, hydrogen isotopes, and all associated 2σ uncertainties as well as saturation pressure estimates.

Data S2. (separate file)
Diffusion exchange model parameter testing and results.

Data S3. (separate file)
Sample descriptions.

Data S4. (separate file)
Electron microprobe major element analysis conditions and precision on standard analyses.

Data S5. (separate file)
Calculation for adding diffusion time from experimental temperature ramp-up period, after (83).

REFERENCES

1. R. L. Christiansen, *The Quaternary and Pliocene Yellowstone Plateau Volcanic Field of Wyoming, Idaho, and Montana* (US Geological Survey, 2001).

2. R. A. Bailey, G. B. Dalrymple, M. A. Lanphere, Volcanism, structure, and geochronology of Long Valley Caldera, Mono County, California. *J. Geophys.* **81**, 725–744 (1976).

3. S. M. Bower, A. W. Woods, Control of magma volatile content and chamber depth on the mass erupted during explosive volcanic eruptions. *J. Geophys. Res.* **102**, 10273–10290 (1997).

4. R. von Glasow, N. Bobrowski, C. Kern, The effects of volcanic eruptions on atmospheric chemistry. *Chem. Geol.* **263**, 131–142 (2009).

5. P. J. Wallace, Volatiles in subduction zone magmas: Concentrations and fluxes based on melt inclusion and volcanic gas data. *J. Volcanol. Geotherm. Res.* **140**, 217–240 (2005).

6. A. M. Shaw, E. H. Hauri, M. D. Behn, D. R. Hilton, C. G. Macpherson, J. M. Sinton, Long-term preservation of slab signatures in the mantle inferred from hydrogen isotopes. *Nat. Geosci.* **5**, 224–228 (2012).

7. P. J. Wyllie, Magmas and volatile components. *Am. Mineral.* **64**, 469–500 (1979).

8. L. Caricchi, T. E. Sheldrake, J. Blundy, Modulation of magmatic processes by CO_2 flushing. *Earth Planet. Sci. Lett.* **491**, 160–171 (2018).

9. G. Moore, Interpreting H_2O and CO_2 contents in melt incusions: Constraints from solubility experiments and modeling. *Rev. Mineral. Geochem.* **69**, 333–361 (2008).

10. P. J. Wallace, A. T. Anderson, A. M. Davis, Gradients in H_2O, CO_2, and exsolved gas in a large-volume silicic magma system: Interpreting the record preserved in melt inclusions from the Bishop Tuff. *J. Geophys. Res. Solid Earth* **104**, 20097–20122 (1999).

11. P. J. Wallace, T. Plank, M. Edmonds, E. H. Hauri, *Volatiles in Magmas* (Elsevier Inc., ed. 2, 2015).

12. P. J. Wallace, V. S. Kamenetsky, P. Cervantes, Melt inclusion CO_2 contents, pressures of olivine crystallization, and the problem of shrinkage bubbles. *Am. Mineral.* **100**, 787–794 (2015).

13. Z. Qin, F. Lu, A. T. Anderson, Diffusive reequilibration of melt and fluid inclusions. *Am. Mineral.* **77**, 565–576 (1992).

14. L. V. Danyushevsky, F. N. Della-Pasqua, S. Sokolov, Re-equilibration of melt inclusions trapped by magnesian olivine phenocrysts from subduction-related magmas: Petrological implications. *Contrib. Mineral. Petrol.* **138**, 68–83 (2000).

15. C. E. Bucholz, G. A. Gaetani, M. D. Behn, N. Shimizu, Post-entrapment modification of volatiles and oxygen fugacity in olivine-hosted melt inclusions. *Earth Planet. Sci. Lett.* **374**, 145–155 (2013).

16. Y. Chen, A. Provost, P. Schiano, N. Cluzel, The rate of water loss from olivine-hosted melt inclusions. *Contrib. Mineral. Petrol.* **162**, 625–636 (2011).

17. G. A. Gaetani, J. A. O'Leary, N. Shimizu, C. E. Bucholz, M. Newville, Rapid reequilibration of H_2O and oxygen fugacity in olivine-hosted melt inclusions. *Geology* **40**, 915–918 (2012).

18. Y. Zhang, G. Gaetani, A. Pamukcu, B. Monteleone, L. Saper, Experimental investigation of hydrogen isotope fractionation during hydration of olivine-hosted melt inclusions: Implications for D/H in Baffin Island picrites. *Earth Planet. Sci. Lett.* **647**, 119052 (2024).

19. D. J. Rasmussen, P. R. Kyle, P. J. Wallace, K. W. W. Sims, G. A. Gaetani, E. H. Phillips, Understanding degassing and transport of CO_2-rich alkalic magmas at Ross Island, Antarctica using olivine-hosted melt inclusions. *J. Petrol.* **58**, 841–862 (2017).

20. M. J. Severs, T. Azbej, J. B. Thomas, C. W. Mandeville, R. J. Bodnar, Experimental determination of H_2O loss from melt inclusions during laboratory heating: Evidence from Raman spectroscopy. *Chem. Geol.* **237**, 358–371 (2007).

21. M. C. Jollands, B. Ellis, P. M. E. Tollan, O. Müntener, An eruption chronometer based on experimentally determined H-Li and H-Na diffusion in quartz applied to the Bishop Tuff. *Earth Planet. Sci. Lett.* **551**, 116560 (2020).

22. M. L. Myers, P. J. Wallace, C. J. N. Wilson, Inferring magma ascent timescales and reconstructing conduit processes in explosive rhyolitic eruptions using diffusive losses of hydrogen from melt inclusions. *J. Volcanol. Geotherm. Res.* **369**, 95–112 (2019).

23. K. S. Befus, K. J. Walowski, R. L. Hervig, J. T. Cullen, Hydrogen isotope composition of a large silicic magma reservoir preserved in quartz-hosted glass inclusions of the Bishop Tuff plinian eruption. *Geochem. Geophys. Geosyst.* **21**, e2020GC009358 (2020).

24. R. L. Nielsen, P. J. Michael, R. Sours-Page, Chemical and physical indicators of compromised melt inclusions. *Geochim. Cosmochim. Acta* **62**, 831–839 (1998).

25. A. J. R. Kent, Met inclusions in basaltic and related volcanic rocks. *Rev. Mineral. Geochem.* **69**, 273–331 (2008).

26. R. Thomas, J. D. Webster, W. Heinrich, Melt inclusions in pegmatite quartz: Complete miscibility between silicate melts and hydrous fluids at low pressure. *Contrib. Mineral. Petrol.* **139**, 394–401 (2000).

27. C. N. Mercer, A. H. Hofstra, T. I. Todorov, J. Roberge, A. Burgisser, D. T. Adams, M. Cosca, Pre-eruptive conditions of the hideaway park topaz rhyolite: Insights into metal source and evolution of magma parental to the henderson porphyry molybdenum deposit, Colorado. *J. Petrol.* **56**, 645–679 (2014).

28. T. R. Benson, M. A. Coble, J. J. Rytuba, G. A. Mahood, Lithium enrichment in intracontinental rhyolite magmas leads to Li deposits in caldera basins. *Nat. Commun.* **8**, 270 (2017).

29. C. M. Skirius, J. W. Peterson, A. T. Anderson, Homogenizing rhyolitic glass inclusions from the Bishop Tuff. *Am. Mineral.* **75**, 1381–1398 (1990).

30. J. R. Bodnar, J. J. Student, Melt inclusions in plutonic rocks: Petrography and microthermometry. *Melt Inclusions Plutonic Rocks* **36**, 1–25 (2006).

31. L. L. Colombini, C. F. Miller, G. A. R. Gualda, J. L. Wooden, J. S. Miller, Sphene and zircon in the Highland Range volcanic sequence (Miocene, southern Nevada, USA): Elemental

partitioning, phase relations, and influence on evolution of silicic magma. *Mineral. Petrol.* **102**, 29–50 (2011).

32. B. M. Wallrich, C. F. Miller, G. A. R. Gualda, J. S. Miller, N. H. Hinz, J. E. Faulds, Volcano-pluton connection: Perspectives on material and process linkages, Searchlight pluton and Highland Range volcanic sequence, Nevada, USA. *Earth Sci. Rev.* **238**, 104361 (2023).

33. G. A. R. Gualda, C. F. Miller, B. M. Wallrich, The rhyolite factory: Insights from rhyolite-MELTS geobarometry of plutonic rocks and associated volcanics. *J. Petrol.* **65**, egae123 (2024).

34. J. E. Faulds, E. L. Olson, S. S. Harlan, W. C. McIntosh, Miocene extension and fault-related folding in the Highland Range, southern Nevada: A three-dimensional perspective. *J. Struct. Geol.* **24**, 861–886 (2002).

35. L. L. Colombini, "Mid-Miocene rhyolite sequence, Highland Range, NV: Record of magma evolution and eruption from the Searchlight pluton magma chamber," thesis, Vanderbilt University (2009).

36. A. S. Pamukçu, S. M. Hickernell, M. P. Eddy, B. Schoene, T. Steiner-Leach, Geology constrains the diffusivity of Ti in quartz and crystallization timescales of high-silica magmas in the Searchlight Magmatic System (NV, USA). *Earth Planet. Sci. Lett.* **666**, 119437 (2025).

37. M. P. Eddy, A. Pamukçu, B. Schoene, T. Steiner-Leach, E. A. Bell, Constraints on the timescales and processes that led to high-SiO_2 rhyolite production in the Searchlight pluton, Nevada, USA. *Geosphere* **18**, 1000–1019 (2022).

38. J. J. Student, R. J. Bodnar, Synthetic fluid inclusions XIV: Coexisting silicate melt and aqueous fluid inclusions in the haplogranite–H_2O–NaCl–KCl system. *J. Petrol.* **40**, 1509–1525 (1999).

39. L. R. Moore, E. Gazel, R. Tuohy, A. S. Lloyd, R. Esposito, M. Steele-MacInnis, E. H. Hauri, P. J. Wallace, T. Plank, R. J. Bodnar, Bubbles matter: An assessment of the contribution of vapor bubbles to melt inclusion volatile budgets. *Am. Mineral.* **100**, 806–823 (2015).

40. A. S. Lloyd, T. Plank, P. Ruprecht, E. H. Hauri, W. Rose, Volatile loss from melt inclusions in pyroclasts of differing sizes. *Contrib. Mineral. Petrol.* **165**, 129–153 (2013).

41. Z. Zajacz, J. J. Hanley, C. A. Heinrich, W. E. Halter, M. Guillong, Diffusive reequilibration of quartz-hosted silicate melt and fluid inclusions: Are all metal concentrations unmodified? *Geochim. Cosmochim. Acta* **73**, 3013–3027 (2009).

42. E. J. F. Mutch, M. E. Newcombe, J. F. Rudge, 3D diffusion of water in melt-inclusion-bearing olivine phenocrysts. *Geochem. Geophys. Geosyst.* **25**, e2023GC011365 (2024).

43. V. S. Kamenetsky, L. V. Danyushevsky, Metals in quartz-hosted melt inclusions: Natural facts and experimental artifacts. *Am. Mineral.* **90**, 1674–1678 (2005).

44. Y. Li, A. Audétat, L. Lerchbaumer, X. L. Xiong, Rapid Na, Cu exchange between synthetic fluid inclusions and external aqueous solutions: Evidence from LA-ICP-MS analysis. *Geofluids* **9**, 321–329 (2009).

45. L. Lerchbaumer, A. Audétat, High Cu concentrations in vapor-type fluid inclusions: An artifact? *Geochim. Cosmochim. Acta* **88**, 255–274 (2012).

46. B. Rottier, H. Rezeau, V. Casanova, K. Kouzmanov, R. Moritz, K. Schlöglova, M. Wälle, L. Fontboté, Trace element diffusion and incorporation in quartz during heating experiments. *Contrib. Mineral. Petrol.* **172**, 1–20 (2017).

47. D. J. Cherniak, E. B. Watson, D. A. Wark, Ti diffusion in quartz. *Chem. Geol.* **236**, 65–74 (2007).

48. A. A. Iveson, J. D. Webster, M. C. Rowe, O. K. Neill, Fluid-melt trace-element partitioning behaviour between evolved melts and aqueous fluids: Experimental constraints on the magmatic-hydrothermal transport of metals. *Chem. Geol.* **516**, 18–41 (2019).

49. B. S. Ellis, D. Szymanowski, T. Magna, J. Neukampf, R. Dohmen, O. Bachmann, P. Ulmer, M. Guillong, Post-eruptive mobility of lithium in volcanic rocks. *Nat. Commun.* **9**, 2–10 (2018).

50. M. C. Jollands, P. M. E. Tollan, L. P. Baumgartner, O. Müntener, Hydrogen diffusion mechanisms in quartz: Insights from H–Li, ^{2}H–H and ^{2}H–H–Li exchange experiments. *Mineral. Mag.* **86**, 112–126 (2022).

51. J. B. Lowenstern, G. A. Mahood, M. L. Rivers, S. R. Sutton, Evidence for extreme partitioning of copper into a magmatic vapor phase. *Science* **252**, 1405–1409 (1991).

52. D. R. Baker, The fidelity of melt inclusions as records of melt composition. *Contrib. Mineral. Petrol.* **156**, 377–395 (2008).

53. A. T. Anderson, "Chapter 14: An introduction to melt (glass and crystals) inclusions," in *Fluid Inclusions: Analysis and Interpretation* (Mineralogical Association of Canada, 2003), pp. 369–380.

54. C. A. Bachl, C. F. Miller, J. S. Miller, J. E. Faulds, Construction of a pluton: Evidence from an exposed cross section of the Searchlight pluton, Eldorado Mountains, Nevada. *GSA Bull.* **113**, 1213–1228 (2001).

55. E. Hauri, SIMS analysis of volatiles in silicate glasses, 2: Isotopes and abundances in Hawaiian melt inclusions. *Chem. Geol.* **183**, 115–141 (2002).

56. H. Ni, Y. Zhang, H_2O diffusion models in rhyolitic melt with new high pressure data. *Chem. Geol.* **250**, 68–78 (2008).

57. K. J. Walowski, P. J. Wallace, E. H. Hauri, I. Wada, M. A. Clynne, Slab melting beneath the Cascade Arc driven by dehydration of altered oceanic peridotite. *Nat. Geosci.* **8**, 404–408 (2015).

58. P. J. Wallace, J. Dufek, A. T. Anderson, Y. Zhang, Cooling rates of Plinian-fall and pyroclastic-flow deposits in the Bishop Tuff: Inferences from water speciation in quartz-hosted glass inclusions. *Bull. Volcanol.* **65**, 105–123 (2003).

59. M. L. Frezzotti, Silicate-melt inclusions in magmatic rocks: Applications to petrology. *Lithos* **55**, 273–299 (2001).

60. M. Portnyagin, R. Almeev, S. Matveev, F. Holtz, Experimental evidence for rapid water exchange between melt inclusions in olivine and host magma. *Earth Planet. Sci. Lett.* **272**, 541–552 (2008).

61. A. S. Pamukcu, M. S. Ghiorso, G. A. R. Gualda, High-Ti, bright-CL rims in volcanic quartz: A result of very rapid growth. *Contrib. Mineral. Petrol.* **171**, 105 (2016).

62. J. H. Seo, C. A. Heinrich, Selective copper diffusion into quartz-hosted vapor inclusions: Evidence from other host minerals, driving forces, and consequences for Cu-Au ore formation. *Geochim. Cosmochim. Acta* **113**, 60–69 (2013).

63. P. M. Harris, C. E. Waring, Diffusion of lithium ions through quartz in an electric field. *J. Phys. Chem.* **41**, 1077–1085 (1937).

64. R. D. Shannon, Revised effective ionic radii and systematic studies of interatomic distances in halides and chalcogenides. *Acta Crystallogr. A* **32**, 751–767 (1976).

65. M. C. Jollands, M. Blanchard, E. Balan, Structure and theoretical infrared spectra of OH defects in quartz. *Eur. J. Mineral.* **32**, 311–323 (2020).

66. S. Yoshimura, M. Nakamura, Chemically driven growth and resorption of bubbles in a multivolatile magmatic system. *Chem. Geol.* **276**, 18–28 (2010).

67. A. G. Simakin, V. N. Devyatova, A. A. Shiryaev, Theoretical and experimental modeling of local scale CO_2 flushing of hydrous rhyolitic magma. *Russ. J. Earth Sci.* **23**, ES6007 (2023).

68. J. M. Castro, I. N. Bindeman, H. Tuffen, C. Ian Schipper, Explosive origin of silicic lava: Textural and δD-H_2O evidence for pyroclastic degassing during rhyolite effusion. *Earth Planet. Sci. Lett.* **405**, 52–61 (2014).

69. D. Giordano, J. K. Russell, D. B. Dingwell, Viscosity of magmatic liquids: A model. *Earth Planet. Sci. Lett.* **271**, 123–134 (2008).

70. J. E. Gardner, J. D. Webster, The impact of dissolved CO_2 on bubble nucleation in water-poor rhyolite melts. *Chem. Geol.* **420**, 180–185 (2016).

71. H. M. Gonnermann, Magma fragmentation. *Annu. Rev. Earth Planet. Sci.* **43**, 431–458 (2015).

72. A. Brookfield, M. Cassidy, G. Weber, R. G. Popa, O. Bachmann, M. J. Stock, Magmatic volatile content and the overpressure ‘sweet spot’: Implications for volcanic eruption triggering and style. *J. Volcanol. Geotherm. Res.* **444**, 107916 (2023).

73. C. Jorgenson, L. Caricchi, M. Chiaradia, M. Ágreda-López, G. Giordano, Rapid accumulation and ascent precedes caldera forming eruption of low viscosity magma. *Contrib. Mineral. Petrol.* **179**, 16 (2024).

74. G. F. Cooper, C. J. N. Wilson, M. A. Millet, J. A. Baker, E. G. C. Smith, Systematic tapping of independent magma chambers during the 1Ma Kidnappers supereruption. *Earth Planet. Sci. Lett.* **313-314**, 23–33 (2012).

75. L. J. Harmon, G. A. R. Gualda, D. M. Gravley, S. L. Smithies, C. D. Deering, The Whakamaru magmatic system (Taupō Volcanic Zone, New Zealand), part 1: Evidence from tephra deposits for the eruption of multiple magma types through time. *J. Volcanol. Geotherm. Res.* **445**, 107966 (2024).

76. E. J. Swallow, C. J. N. Wilson, M. L. Myers, P. J. Wallace, K. S. Collins, E. G. C. Smith, Evacuation of multiple magma bodies and the onset of caldera collapse in a supereruption, captured in glass and mineral compositions. *Contrib. Mineral. Petrol.* **173**, 33 (2018).

77. K. V. Cashman, G. Giordano, Calderas and magma reservoirs. *J. Volcanol. Geotherm. Res.* **288**, 28–45 (2014).

78. J. E. Faulds, J. Bell, E. Olson, “Geologic map of the Nelson SW quadrangle, Clark County, Nevada,” Nevada Bureau of Mines and Geology Map 134 (Nevada Bureau of Mines and Geology, 2002).

79. T. W. Sisson, T. L. Grove, Experimental investigations of the role of H_2O in calc-alkaline differentiation and subduction zone magmatism. *Contrib. Mineral. Petrol.* **113**, 143–166 (1993).

80. T. Hanyu, J. Yamamoto, K. Kimoto, K. Shimizu, T. Ushikubo, Determination of total CO_2 in melt inclusions with shrinkage bubbles. *Chem. Geol.* **557**, 119855 (2020).

81. R. Macdonald, R. L. Smith, “Chemistry of the subalkalic silicic obsidians” (Rep. No. 1523, US Gov. Print. Off., 1992).

82. A. J. Padilla, G. A. R. Gualda, Crystal-melt elemental partitioning in silicic magmatic systems: An example from the Peach Spring Tuff high-silica rhyolite, Southwest USA. *Chem. Geol.* **440**, 326–344 (2016).

83. S. S. Rout, B. C. Schmidt, G. Wörner, Constraints on non-isothermal diffusion modeling: An experimental analysis and error assessment using halogen diffusion in melts. *Am. Mineral.* **105**, 227–238 (2020).

84. M. Cassidy, M. Manga, K. Cashman, O. Bachmann, Controls on explosive-effusive volcanic eruption styles. *Nat. Commun.* **9**, 2839 (2018).

85. M. S. Ghiorso, G. A. R. Gualda, An H_2O–CO_2 mixed fluid saturation model compatible with rhyolite-MELTS. *Contrib. Mineral. Petrol.* **169**, 53 (2015).

86. K. Iacovino, S. Matthews, P. E. Wieser, G. M. Moore, F. Bégué, VESIcal part I: An open-source thermodynamic model engine for mixed volatile (H_2O-CO_2) solubility in silicate melts. *Earth Space Sci.* **8**, e2020EA001584 (2021).

87. S. C. Walter, J. M. Castro, VolcDeGas: A program for modelling hydrogen isotope fractionation during degassing of rhyolitic melts. *Volcanica* **3**, 155–168 (2020).

88. A. S. Pamukçu, G. A. R. Gualda, M. L. Rivers, Quantitative 3D petrography using x-ray tomography 4: Assessing glass inclusion textures with propagation phase-contrast tomography. *Geosphere* **9**, 1704–1713 (2013).
